# Morphological and Electrical Properties of Proteinoid–Actin Networks

**DOI:** 10.1021/acsomega.4c10488

**Published:** 2025-01-27

**Authors:** Panagiotis Mougkogiannis, Andrew Adamatzky

**Affiliations:** Unconventional Computing Laboratory, University of the West of England, Bristol BS16 1QY, U.K.

## Abstract

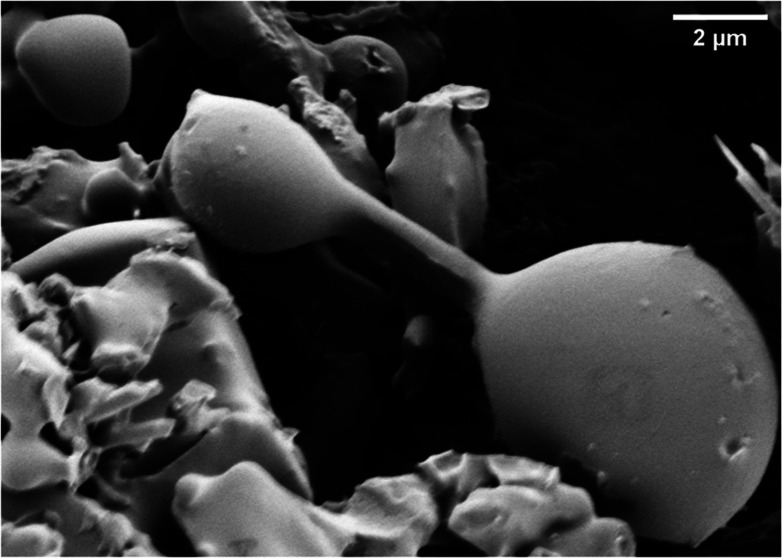

Proteinoids, or thermal proteins, are produced by heating amino acids. Proteinoids form hollow microspheres in water. The microspheres produce oscillation of electrical potential. Actin is a filament-forming protein responsible for communication, information processing and decision making in eukaryotic cells. We synthesize randomly organized networks of proteinoid microspheres spanned by actin filaments and study their morphology and electrical potential oscillatory dynamics. We analyze proteinoid–actin networks’ responses to electrical stimulation. The signals come from logistic maps, the Lorenz attractor, the Rossler oscillator, and the FitzHugh–Nagumo system. We show how the networks attenuated the signals produced by these models. We demonstrate that emergent logical patterns derived from oscillatory behavior of proteinoid–actin networks show characteristics of Boolean logic gates, providing evidence for the computational ability to combine different components through architectural changes in the dynamic interface. Our experimental laboratory study paves a base for generation of proto-neural networks and implementation of neuromorphic computation with them.

## Introduction

Thermal proteins—proteinoids—are generated by subjecting amino acids to elevated temperatures until reaching their melting point, thereby initiating polymerization to form polymeric chains. Polymerization occurs within the temperature range of 160–200 °C, without the presence of a solvent, initiator, or catalyst, and in an inert atmosphere. Amino acids with trifunctional properties, such as glutamic or aspartic acid or lysine, undergo cyclization at high temperatures, functioning as solvents and initiators for the polymerization of other amino acids.^1,2^ This uncomplicated thermal condensation reaction allows the production of proteinoids with either acidic or basic characteristics. A proteinoid can be expanded in an aqueous solution at moderate temperatures (approximately 50 °C), resulting in the formation of microspheres.^2^ These microspheres are typically hollow and often contain an aqueous solution. The proteinoid microspheres maintain a steady state membrane potential 20 to 70 mV without any stimulating current. Some microspheres in the population display the opposite polarization steadily.^3^ Electrical membrane potentials, oscillations, and action potentials are observed in the microspheres impaled with microelectrodes. These microspheres exhibit action-potential like spikes. The electrical activity of the microspheres also includes spontaneous bursts of electrical potential (flip-flops), and miniature potential activities at flopped phases.^4^ The electrical properties of behavior of proteinoids microspheres inspired Sydney Fox and colleagues in early 1990s to propose these structure as proto-neurons, replacements of Oparin used coacervate protocols.^5^

Actin is a type of cytoskeletal protein that has the ability to form filamentous networks.^6−8^ Actin is a protein that is abundantly expressed in all eukaryotic cells.^9^ It plays a crucial role in cellular functions by forming an intracellular scaffold, actuators, and pathways for information transfer and processing. There is supporting evidence indicating that actin may serve as a conduit for electrical potential and ionic waves,^10^ as well as participating in quantum protein transitions,^11,12^ alongside its established roles in mechanical force transmission and signaling cascades. Both experimental observations and modeling efforts have demonstrated the ability of actin to function as biowires capable of conducting ionic waves.^10,13−18^ Actin filaments, being polyelectrolytes surrounded by counterions, possess the capability to transmit signals or sustain ionic conductances.^10,19^

Based on the above, we have proto-neurons made of proteinoid microspheres and proto-axons/dendrites made of actin filaments. Therefore, we can make a proto-neural network. Actin filaments are engineered through controlled polymerization inside the proteinoid microspheres, rather than emerging spontaneously. A key idea is to design and prototype in laboratory conditions a proto-neural network where proteinoid microspheres are spanned by actin filaments. We believe that endogenous and induced electrical oscillations of proteinoids can be transferred via actin filaments, thus allow information propagation, processing and computation.

Biological organisms utilize oscillatory dynamics at many levels to process information, perform computations, and exert control.^20−27^ Networks consisting of interconnected oscillators enable a wide range of tasks, including the regulation of biological cycles, encoding of brain information, and facilitating movement.^28−32^ Transferring these abilities to artificial systems continues to be a significant obstacle. Utilizing the intricate biochemical complexity seen in live systems, bio-inspired methods offer promising avenues for exploiting oscillatory dynamics.^33−35^ Composite materials that combine biological molecules with synthetic structures offer a fascinating foundation for building oscillator networks.^36−39^

Previous research into the integration of synthetic architectures with cytoskeletal assemblies has demonstrated the possibility for coordinated behaviors useful in computational applications.^40−42^ Notably, the networks generated by cytoskeleton polymer actin demonstrate the realization of fundamental logic gates, highlighting the assemblies’ ability to implement advanced Boolean logic.^43−46^ Beyond simple electrical coupling, reaction–diffusion processes enable communication channels that take advantage of actomyosin contractility and diffusion, opening up new possibilities for the development of unconventional computing systems.^47−49^ These studies highlight bio-hybrid materials’ diverse and dynamic capabilities in information processing.^50,51^ However, it is worth noting that the majority of these studies have exclusively concentrated on a single cytoskeletal component, ignoring the different cooperation lengths and timelines that are inherent in biological systems. Future research should address this limit. It should seek a better understanding of the synergistic interactions of various cytoskeletal elements in novel computing.

**Figure 1:**
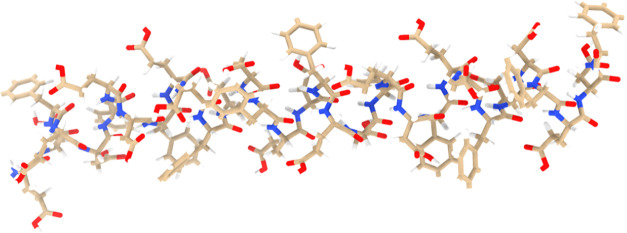
Molecular modeling of a thermal proteinoid peptide consisting of l–glutamic acid, l–phenylalanine, and l–aspartic acid. The proteinoid is visualized with 11 amino acid residues showing backbone ribbon structure along with element color–coded atoms (Gray—Hydrogen, Red—Oxygen, Blue—Nitrogen, Brown—Carbon). By designing the proteinoid model and minimizing its energy to −1442.88 kJ/mol via the ChimeraX modeling system, a stable conformer was confirmed.^52^ When incorporated as a soluble network with cytoskeletal components, these nanoscale proteinoid confirmations likely underpin the electron mobility, capacitance, and self-assembly profiles that initiate the emergence of complex oscillatory phenotypes at larger scales.

**Figure 2:**
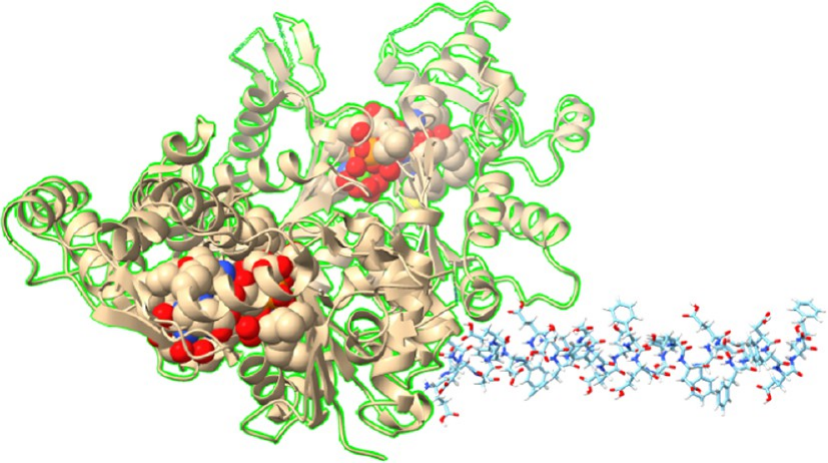
Model depicts the integration of a rabbit muscle actin filament with a thermal l–Glu:l–Phe:l–Asp proteinoid. The actin structure shows the distinctive helical arrangement of globular actin subunits. One end of the filament is closely associated with the proteinoid membrane interface, indicating potential binding sites such as surface carboxyl groups that allow bioconjugation. For clarity, the structures include backbone traces as well as element color-coded atoms for both components. Understanding interfacial structural features at the proteinoid–actin interface can aid in linking conformational alignment to productive conduction channels activated by the composite. Generally, multi-scale views highlight the architectural integration of biological cytoskeletal components with synthetic protocell-like compartments in order to construct hybrid bio-materials for unconventional computing applications. The structure of the Actin filament was found and subsequently modified based on data from.^53^

The integration of proteinoid–actin networks will provide a convincing framework for combining biological and abiotic components to produce functionalities that are beyond the capabilities of each alone. In addition to experimental implementations of bio-inspired models, these hybrid systems provide a two-way flow of knowledge between theoretical frameworks and direct empirical data. As observed in the molecular modeling of a proteinoid peptide containing l–glutamic acid, l–phenylalanine, and l–aspartic acid (Figure 1), energy optimization reveals significant backbone confirmation and secondary structuring. The proteinoid–actin network we have developed combines proteinoid microspheres that have undergone thermal processing with rabbit cytoskeletal filaments (Figure 2). This innovative approach brings together two important aspects of cellular architecture-localized compartmentalization and networked connectivity.^54^ The proposed connections are expected to involve carboxyl groups on proteinoids binding with amino groups on actin, resulting in strong amide bonds by carbodiimide bioconjugation.^55^

## Methods and Materials

### Preparation of Proteinoid–Actin Networks

Actin Binding Protein Spin-Down Assay Biochem Kit comprising rabbit skeletal muscle actin was purchased from Cytoskeleton, Inc. The amino acids l–Aspartic acid, l–Phenylalanine, and l–Glutamic acid were acquired from Sigma-Aldrich and used without additional purification. Upon procurement, the thermal polycondensation technique was implemented for proteinoids synthesis as described by Mougkogiannis et al.^56^ Equimolar mixtures of l–Aspartic acid, l–Phenylalanine and l–Glutamic acid were heated at 180 °C for 30 min under nitrogen atmosphere with constant stirring. Alongside the amino acid mixtures, rabbit skeletal muscle actin was introduced in a 1% w/w ratio during the thermal polymerization process. The proteinoids formed were separated from excess reactants via lyophilization and stored at room temperature for subsequent analyses. The proteinoid–actins’ morphology was characterized by capturing scanning electron micrographs using a Quanta 650 microscope. Prior to SEM imaging, the samples were gold-coated to enhance their conductivity and optimize imaging quality.

### Recording of Electrical Activity

Subdermal electrodes made of platinum–iridium coated stainless steel (manufactured by Spes Medica S.r.l) were inserted into proteinoid–actin samples, with a spacing of roughly 10 mm between them. The Pico Technology ADC-24 data logger, with its high resolution and 24-bit analog–digital converter, accurately recorded the activity of electrodes. The study utilized an Ossila Instruments manual potentiostat (Model: T2006A) to conduct open potentiometry examination. The approved measurement protocols were followed during the experiments.

### Probing Proteinoid–Actin Networks with Quasi-Chaotic Inputs

In this section, we investigate how our biohybrid networks process complex, unpredictable signals. We can test the networks using chaotic patterns from math models (logistic maps, Lorenz attractors, and Rossler systems). This will evaluate their ability to process diverse inputs. This approach helps us understand whether these networks can reliably process irregular signals, similar to biological neurons. To evaluate proteinoid–actin networks’ response to a wide range of electrical stimuli we decided to probe them with quasi-chaotic sequences of voltage values generated by logistic maps and Lorenz attractors.

#### Logistic Maps

Utilizing discrete logistic maps as a framework for generating controlled chaotic voltage patterns offers a clear and approachable starting point before delving into the more complex realm of proteinoid compositions in higher dimensions. The logistic map, renowned for its one-dimensional discrete bifurcation dynamics, adheres to the difference equation
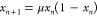
1The logistic map exhibits diverse dynamic phenomena such as stable points, sites of divergence, chaotic patterns, periodic intervals, and complexities in development that depend on the growth rate factor μ.

To stimulate proteinoid–actin networks with logistics maps we converted value *x*() to voltage as follows. We use numbers between 0 and 1 (fractions) because the logistic map is mathematically defined to operate in this range. To generate the desired waveforms, we iterate the Logistic difference eq 10,000 times, starting from randomly selected starting values between 0 and 1. This process produces pseudorandom numbers that exhibit sensitivity to these initial conditions. By setting the bifurcation parameter, μ, a value of 3.8, we ensure the appearance of fully developed chaos that extends across the entire unit interval. This has been confirmed by our analysis using Lyapunov metrics. In order to convert these disordered fractions into voltage signals, we apply a linear scaling process that adjusts them to predetermined upper and lower limits (−500 to +500 mV).

Instead of concentrating on tracking individual waveforms, we employ an input–output analytic approach that compares statistical features. This integration between mathematically constructed randomness and biophysical stimulation protocols enables the control of emergent bio-electronic systems.

The input voltages we provide systematically adjust the μ factor, which in turn affects the responses generated by the chemical reaction network inside the gate. By alternating between low and high values of μ, the system has the ability to provide either consistent fixed concentration outputs or complex signals exhibiting significant fluctuations. The integration of feedback loops that redirect output states back into the system, resulting in the dynamic adjustment of growth parameters, enables the development of autonomous or self-contained chaotic circuits. These circuits can create random-like numbers. But, their main purpose here is to make complex, deterministic voltage patterns. They help us understand how our proteinoid–actin networks process and respond to irregular, unpredictable signals. We have assessed the Lyapunov exponent, which quantifies the level of chaos in a system, using different increments of μ. Furthermore, besides quantitatively confirming the existence of deterministic chaos through positive Lyapunov values, it also emphasizes the complex reorganization of microenvironments when they are significantly deviated from homeostasis. Examining higher-dimensional systems using continuous dynamics provides a more accurate portrayal of the collaborative interactions among components of a system.

Chaotic oscillators offer a means to computationally analyze fluidic phenomena through controllable and dynamic transformations.^57−59^ These oscillators are math models. They generate unpredictable but deterministic signal patterns, like complex fluid flows in nature. Using such oscillators, we can study how our proteinoid–actin networks react to various irregular inputs. This will help us understand their information processing. We utilize a Proteinoid–Actin Baker’s Map that incorporates stretching and folding principles to mathematically simulate spiking.^60^ This paradigm of chaos serves as a method of validation by connecting to the experimental proteinoid–cytoskeletal system. By monitoring the movement of particles during repeated fold–stretch cycles induced by varying input voltages, we can accurately measure the degree of chaos and the efficiency of mixing. Lyapunov exponents are used to calculate the sensitivity of a system, while entropy scores provide a measure of global dispersal.^61^ In our experiments, we drive the proteinoid–actin networks with chaotic signals. The networks’ response should show Lyapunov characteristics like those of the input signals. This would mean the system is a consistent signal processor. Lyapunov exponents measure how nearby trajectories diverge in the network’s response. They should correlate with the chaotic properties of the driving signals. This will let us assess how well the network preserves and processes complex input patterns. Testing different input amplitudes helps to find nonlinearity. It also allows for the analysis of mobility vectors. These can link macro-manifestations to microscopic reconfigurations. Nonlinearity has key signatures in the system’s response: (1) Output changes are not proportional to input changes. Doubling the input amplitude does not simply double the output. (2) New frequencies appear in the output that were not in the input signal. (3) The system responds differently to equal-magnitude positive and negative inputs. We look for these indicators when analyzing our proteinoid–actin networks. We examine their responses to different input amplitudes. In general, studying well-known chaotic systems as test models can provide valuable insights into the computational capabilities of emergent proteinoids.

#### Lorentz System

Chaotic dynamics offer rich possibilities for exploring complex signal propagation in engineered biomaterials. Studying chaotic dynamics does more than create random patterns. It helps us to develop materials that process information like biological systems. It also helps in creating sensitive biosensors and adaptive materials. By studying how our proteinoid–actin networks tackles chaos, we can create materials with brain-like processing.^62^ This may lead to new biological computers and smart biomaterials that respond to their environment. This is key for making artificial neural networks. They should mimic the complexity and adaptability of biological systems.^63^ To subject proteinoid–actin networks to chaotic stimulation we adopted the Lorentz system, consisting of three coupled differential equations^64^ first derived to model atmospheric convection^65^
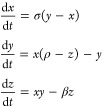
2Here *x*, *y*, and *z* denote system states, while σ, ρ, and β represent empirically derived parameters. The Lorentz framework’s spreading trajectories and topological transitivity in phase space lend rich chaotic dynamics^66^—an intriguing driving stimulus for nonlinear biomaterials. By interfacing Lorentzian waveforms with proteinoid–actin composites, we explore whether microscale cytoskeletal couplings can regulate macroscale input volatility. Quantifying signal transformations via time-series analyses and spectroscopy spotlights the construct’s emergent spatiotemporal filtering response. The algorithmic generation of voltage time series with chaotic volatility is possible by numerically integrating a set of equations known as the Lorenz system.

The parameters of the Lorenz ordinary differential equation were assigned the values σ = 10, β = 8/3, ρ = 28, which are rooted to generate chaotic attractors. The starting state vector was defined as *y*0 = [0; 1; 1.05]. The time evolution was calculated using a fourth-order Runge–Kutta solver with a step size of 0.01 over a duration of 100 s. The chaotic input waveform driving proteinoid–actin dynamical experiments was derived from the *x-*component of the simulated Lorenz trajectory. Similar methodologies have been employed to generate chaotic stimuli with exponential divergence by substituting corresponding vector fields and parameter settings into the integrator schema mentioned above, using versions such as the Rössler attractor. Using an ensemble of computer-generated waveforms from various chaotic systems helps to demonstrate the ubiquity of microscale signal analysis characteristics that are engaged through the bio-composite interface.

Algorithmically generated discrete binary bit streams were used to investigate the integrated bio-interface. The spiking patterns, which are combinations of random 0s and 1s, can be effectively imitated and used for thresholding operations. Using MATLAB’s built-in *rand()* function, binary strings were generated with a resolution of 1 ms, encompassing a time range of 50–100 s. Unlike chaotic signals for Lyapunov exponent calculations, these binary patterns serve a different purpose. They let us test the network’s basic signal processing. We want to see if it can distinguish between discrete states and maintain consistent thresholding behavior. This provides a foundational understanding of the system’s reliability before proceeding to more complex chaotic analysis. The code was seeded to guarantee the reproducibility of pseudo-random sequences. The process of converting into impulse trains entails representing 1’s as a 100 ms increase in channel amplitude to +500 millivolts, while 0s are represented as a 100 ms decrease in channel amplitude to −500 millivolts. This process generates random patterns of stimulation consisting of spikes and periods of silence. These controlled binary inputs are a calibration tool. They establish the system’s baseline response and signal-to-noise ratio. They augment, but are distinct from, the chaotic analysis used for Lyapunov exponent calculations.

In order to quantify sensitivity to initial conditions, Lyapunov exponents calculate the exponential divergence rate in phase space between adjacent trajectories. They were computed by an algorithm adapted from Wolf et al.^61^ implemented in MATLAB. We use two complementary approaches in our experiment. First, we calculate the Lyapunov exponents of our input chaotic signals. This characterizes their inherent complexity. Next, we analyze our proteinoid–actin network’s response to the signals. We do this by measuring the Lyapunov exponents of its output signals. By comparing the Lyapunov exponents of the input and output, we can tell if the network preserves, amplifies, or dampens the chaos in the driving signals. This lets us see how well our biointerface transmits complex time patterns. Placing point pairs above a minimum separation threshold (0.001 V) and separated by a fixed number of steps along the data series (in this case, 50), the procedure selects them iteratively. The ratio of successive divergence magnitudes for qualifying pairs approximates the Lyapunov exponent along local vector lines. Prior to analysis, input and output traces underwent smoothing through the implementation of a Savitzky–Golay FIR filtering (order 2, frame length 15). Assuring numerical instability, the value of ϵ was configured to 10^–5^. These parameters find a balance between data conditioning and intra-model integrity, which is crucial for valid Lyapunov analysis.

#### Rössler Attractor

The Rössler system defines a continuous-time dynamical system exhibiting chaotic oscillations useful for exploring complex biosignals. First studied by Otto Rössler,^67^ it is governed by the set of coupled ordinary differential equations
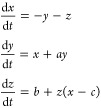
3

Where *x*, *y*, *z* denote system states and *a*, *b*, *c* represent control parameters originally set at *a* = *b* = 0.2, *c* = 5.7 to yield a chaotic attractor.^68^ The system displays outward spiraling trajectories that twist across dimensions—generating continuous broadband oscillations prime for probing bio-inspired interfaces. By combining Rössler waveforms with emergent proteinoid–actin dynamics, we exploit the complexity that arises from both built living materials and intended chaotic systems. The primary goal here is to test how accurately our proteinoid–actin networks can reproduce and process different types of complex input signals. The input is the Rössler system’s spiraling trajectories. They are test signals with known properties. By seeing how well our bionetwork can mimic these patterns, we can assess its potential as a signal processing system. We can also understand its limits in reproducing different dynamic behaviors. This helps us assess whether these networks could potentially serve as biological computing elements. The process of measuring mutual transformations by examining the connections between attractor projections and architectural reconfigurations highlights unconventional paths of computation that exist in both domains.

#### Stimulating Proteinoid–Actin Systems with Output of FitzHugh–Nagumo Model

The FitzHugh–Nagumo model provides a simplified representation of neuronal excitation and propagation dynamics. As originally demonstrated for Hodgkin–Huxley models of action potential generation,^69^ the FitzHugh–Nagumo equations capture essential excitation and recovery processes via coupled fast and slow variables^70^
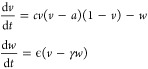
4

Where *v* denotes the fast activation variable, and *w* represents the slow recovery variable. The parameters *a*, *c*, ϵ, and γ dictate excitability thresholds, time scales and other dynamics. Above a critical input current, autonomous oscillations emerge mimicking repetitive neuronal spiking.^71^

Interfacing such model biological oscillators with proteinoid–actin networks could enable insightful investigations into coupled excitable systems across scales. Exploring modalities from electrical to chemical couplings, and relating synchronization motifs to microscopic cytoskeletal rearrangements can spotlight unconventional bio-computation pathways. The rich FitzHugh–Nagumo dynamics, from excitability to birhythmicity, should manifest detectable transformation signatures when interfaced with the integrated biomaterial platform.

## Results

Our research first investigates the diverse morphological properties expressed in self-organized proteinoid–actin composites using microscopic imaging techniques. By “morphological properties,” we mean the physical characteristics visible under a microscope. These include the size, shape, surface texture, and arrangement of the proteinoid microspheres. We also mean the organization and connections of the actin filaments between these structures. These features help us see how the fragments assemble into networks. Following topological characterization, we explore opportunities harnessing these fibrous protein networks to implement two categories of chaotic dynamics systems amenable for unconventional computing: (1) discrete-time systems with discontinuous state updates and (2) continuous-time systems with smooth state evolutions. Both modalities provide rich reconfigurability for complex pattern generation, nonlinear transformations, and logic operations.

### Elucidating Proteinoid–Cytoskeletal Network Morphologies via Scanning Electron Microscopy

**Figure 3:**
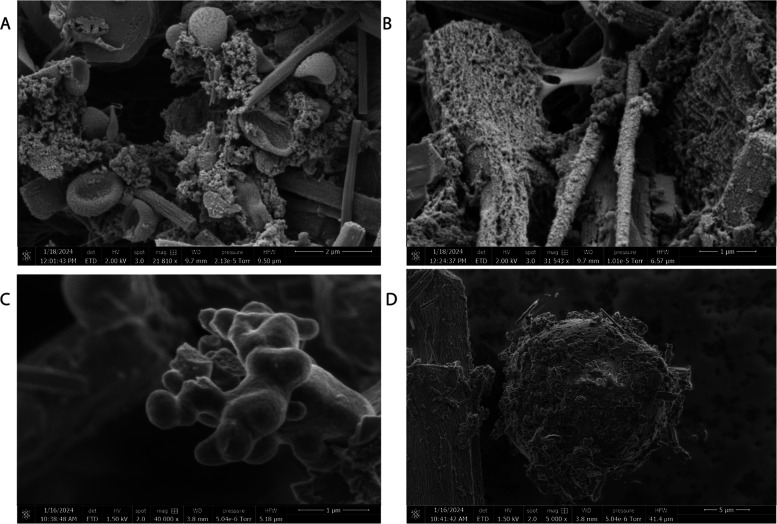
Scanning electron micrographs depict the varied structures observed in l–glutamate–l–phenylalanine-l–aspartate (l–Glu:l–Phe:l–Asp) proteinoids combined with actin filaments during the process of self-assembly. (A) (size bar = 2 μm) shows tiny neuronal ganglion-like structures that exhibit significant roundness and inter-connectedness, mimicking rudimentary cognitive substrates. (B) (with a size bar of 1 μm) exhibits a significant level of intricacy in the branching of the network, showcasing early formations of myelinated architectures. (C) (with a scale bar of 1 μm) shows a complex arrangement of fascicles, which are surrounded by a sheath-like structure. (D) displays proteinoid microspheres of approximately 20 μm in size, observed in the proteinoid–actin suspensions. The scale bar in the image indicates a length of 20 μm. The visualized microstructure motifs collectively display a range of shapes, starting from fragmented nucleation and progressing to more complex oligomers. These structures are created through nonlinear reaction–diffusion processes that determine the arrangement of molecules and their curvature.

The scanning electron micrographs presented here reveal nontrivial alignments of proteinoid architecture when in contact with cytoskeletal filaments. These alignments are observed in various structures, including organized ganglion-forming clusters (Figure 3A), early myelinated branching complexes (Figure 3B), higher order encapsulated fascicular ensembles (Figure 3C), and even large 20 μm spheres (Figure 3D) that are not present in controls.

**Figure 4:**
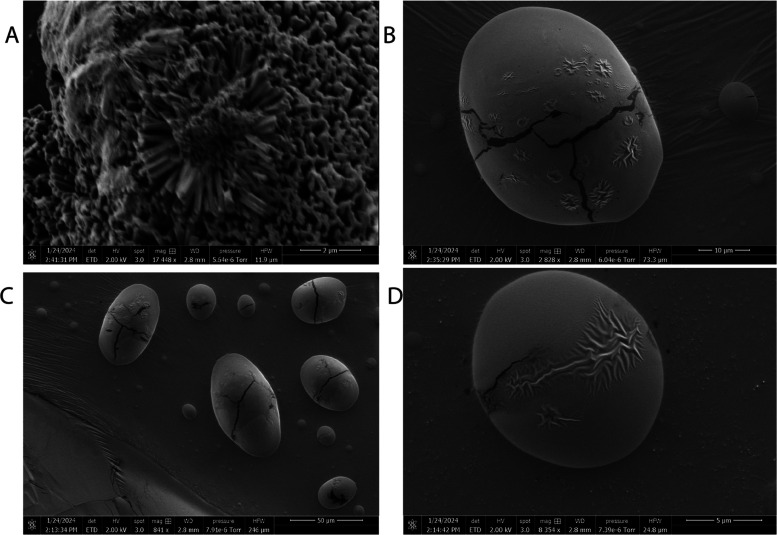
Microspheres formed by the hierarchical organization of the actin cytoskeletal network at many scales. (A) This scanning electron micrograph highlights the intricate nano-structured actin fibers that make up the biosynthetic networks. The scale bar represents a length of 2 μm. (B) Spontaneous formation of microspherical protrusions occurs within the dynamic actin networks, with a diameter of around 10 μm. (C) Surveying the perspective of many microparticles embedded within the continuous fibrous mesh, with a scale bar of 50 μm. (D) A detailed examination at high magnification shows the presence of both smooth and collapsed/buckled spheres. The scale bar represents a length of 5 μm. Overall, complementary imaging techniques provide a clear understanding of the many structures found at the nanoscale level of proteins and the larger spherical assemblies formed by the cytoskeletal component. An important difficulty in understanding the mechanisms of productive signal processing in dynamic biomaterial composites is the correlation of morphological cues across domains with the formation of coordinated excitation and conductivity.

The electron micrographs displayed in Figure 4 demonstrate the complex and dynamic structure of the actin cytoskeletal network. This structure has the ability to spontaneously form spherical micro-protrusions even in the absence of proteinoids. An analysis of the morphological patterns in different figures reveals significant distinctions: actin nanostructures and neighboring microdomains are connected by limited basal attachments. Nevertheless, when actin is combined with proteinoids, it exhibits significant overall connectivity with templated spheres and its surroundings in composite systems. This confirms that when appropriate conjugation conditions are used, large-scale scaffolds are created.

The absence of inherent connections in isolated actin emphasizes that manufactured interactions enable strong interlinking. In the absence of deliberate conjugation procedures, the self-assembly of various biomolecules may be limited to specific interactions rather than forming integrated structures. The previous observations of the large interconnected network and branching connections provide support for our conjugation methods in creating integrated composites of proteinoid and actin.

The present study demonstrates that actin nanostructures create minimum basal attachments intrinsically throughout microdomains, as evidenced by comparing the morphological motifs of the Figures 3 and 4. Nevertheless, actin does exhibit significant overall interconnectedness between self-assembled spheres and their surroundings when combined with proteinoids. This confirms that the use of carbodiimide linkages in engineered conjugation allows for the creation of strong connections at multiple scales.

**Figure 5:**
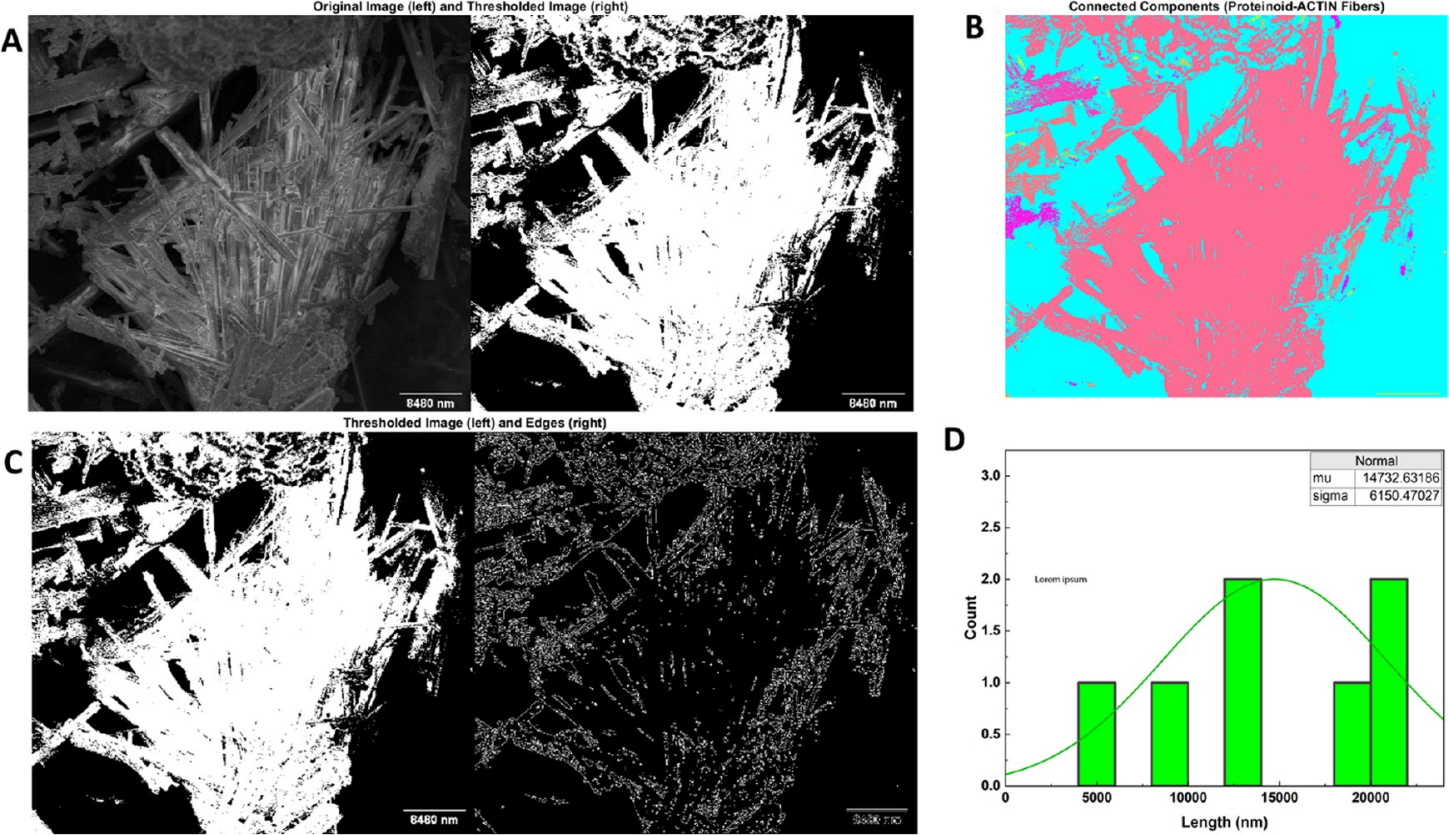
Digital image processing and quantification of proteinoid–actin fiber architectures. (A) Original micrograph compared against threshold-binarized version to highlight detected edges. (B) False-colored connected component labeling employed to distinguish individual fiber regions. (C) A comparison between the binary and Canny edge filter outputs for smoothing and sharpening. (D) A histogram of computed cross-sectional areas that indicates appreciable length variability with a mean of 14,732.6 nm and a standard deviation of 6150.47 nm. The automated image-to-data process enables rapid topological characterization down to single fiber resolution. Correlating morphological, electrical, and microscopic data may help clarify the mechanistic driving factors that link across measurement modalities in the dynamic bio-synthetic network.

Automated image analysis (Figure 5) offer swift quantification of topology, including the segmentation of individual fibers and the measurement of their cross-sectional geometries. The application of thresholding separates fibers from the background prior to performing polygon-bounded linked component labeling, which allows for the identification and numerical size of microstructures. The calculated empirical area distribution demonstrates significant variability at the individual unit level. The average length reaches 14,732.6 nm, while the standard deviations cover similar magnitudes at 6150.5 nm. The question of whether quantified variability is a result of random self-assembly or if it contains meaningful information about the environment’s history from a structural memory standpoint requires further exploration. The observed variations in shape are most likely caused by the fundamental conduction processes occurring at the core. Nevertheless, employing in situ imaging could assist in precisely determining the spatial distribution of hypothesized architectonic motifs that exhibit mixed conducting, insulating, and semi-conducting properties.

### Discrete Logistic Map

The composite l–Glu:l–Phe:l–Asp:Actin proteinoid–cytoskeleton network was analyzed using transient electrical profiling by stimulating proteinoid–actin networks with voltage derived from logistic maps.

A composite system consisting of proteinoid microspheres (l–Glu:l–Phe:l–Asp) combined with actin cytoskeletal filaments, was developed. The electrical properties of this integrated bio-hybrid network were studied using transient stimulus–response analysis. The stimulus particularly featured voltage signals that were algorithmically generated via the chaotic Logistic Map mathematical series. Quantifying the signal processing capacity can be accomplished by analyzing the dynamic proteinoid–actin response to controllably random voltage waveforms obtained from chaotic systems. This approach builds on methods from neuroscience and nonlinear dynamics. Chaotic inputs have been used to study information processing in biological neural networks.^72,73^ Similarly, in neural networks and reservoir computing, chaotic signals help evaluate a system’s computing power.^74^ We can assess the potential of our proteinoid–actin networks as biological computing elements. We will use established principles to do this.

**Figure 6:**
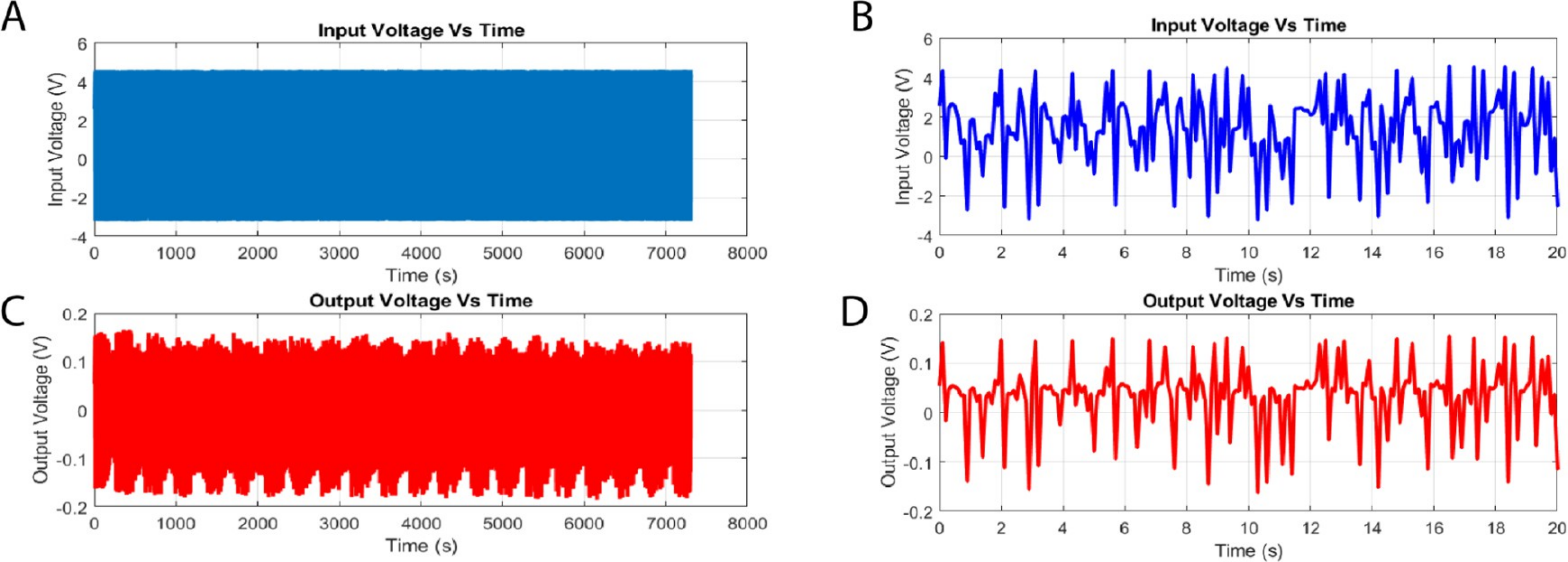
Temporal evolution of the voltage dynamics in the l–Glu:l–Phe:l–Asp: Actin filament system. (A) displays the complete time series of the input voltage plotted against time. The input voltage, shown in (B) for the time interval of 0–20 s, has an average of 1.44 V and a standard variation of 2.07 V. It fluctuates with an estimated alternate frequency of 3.92 Hz. (C, D) Respectively depict the entire series and the 0–20 s interval of the output voltage plotted against time. The output voltage has a mean of 0.02 V and a standard deviation of 0.06 V. The estimated frequency of “peaks per second” is 3.87 Hz. The statistical analysis and distributions of the input and output voltages indicate the presence of inherent diversity and complexity in the active proteinoid–actin network.

This analysis revealed a complex dynamical landscape that is characterized by frequent state fluctuations, phase transitions, and signal transformations. The programmed input voltage signals applied (as shown in Figure 6A) exhibit significant oscillatory patterns, with an average peak magnitude of 1.44 V (standard deviation: 2.07 V) with a dominant frequency of around 3.92 Hz.

On the other hand, the output voltages measured in proteinoid–actin networks (as shown in Figure 6C) demonstrate significantly reduced average values of approximately 0.02 V (with a standard deviation of 0.06 V). However, they still maintain a spectrum profile comparable to the input throughout the observation period.

For example, there is a typical frequency of 3.87 Hz within the first 20-second interval, as depicted in Figure 6D. The presence of a mismatch between the input and output voltage distributions suggests that there is a notable nonlinearity in the conductivity processes of proteinoid–actin. This interpretation follows from basic principles in electrical testing of materials. A linear system would keep proportional relationships between input and output signals. In biological and bioinspired systems, input–output mismatches are common. They occur in ion channels^75^ and protein-based conductors.^76^ The variations indicate voltage-dependent changes in the material’s conductivity. Other protein-based electronic devices show similar nonlinear behavior. In them, the current does not follow Ohm’s law with applied voltage.

The structural network undergoes transitions between quasi-metastable configurations based on the inherent activation of collective variable photochromic and chemomechanical feedback mechanisms, which are currently not operationally connected to applied signals. The observed variations in signaling are most likely caused by changes in the morphological state, which actively control the movement of electrons. Additional in situ microscopy and spectroscopic techniques can be utilized to precisely identify the locations of conductive paths and establish a relationship between the changes in morphology and electrical data.

Our analysis of the chaotic input oscillations and observed outputs from the integrated proteinoid–actin network demonstrates a substantial degree of dynamic modification and signal filtering (see Table 1). In contrast, the average peak voltages of the input logistic map oscillations are 1.44 V with a standard deviation of 2.07 V. The system outputs have significantly lower average voltages of approximately 0.02 V, with a closely controlled standard deviation of 0.06 V. The amplitude may be diminished, but the output frequencies nearly replicate the spectral density of the input signals, notably targeting frequencies around 3.87 and 3.92 Hz respectively.

**Table 1:** Analyses of Chaotic Input Oscillations and Subsequent Output Voltage Responses in an Integrated System Containing Thermally Processed l–Gly:l–Phe:l–Asp Proteinoid and Actin Filaments^a^

	voltage (V)	
metric	input	output
mean	1.44	0.02
std. dev.	2.07	0.06
median	1.81	0.04
max	4.60	0.17
min	–3.24	–0.19
frequency [Hz]	3.919	3.874

a The imposed input pattern follows a discretized logistic map equation with variable degrees of chaos depending on the tunable μ growth parameter. Similarly, the composited proteinoid–actin network exhibits observable electrical modifications such as amplitude suppression and spectrum rearrangement, as seen by dominant frequency modes that mimic the input signals. Such macroscale dynamic adaptations point to widespread morphological reconfigurations occurring within the integrated bio-ionic substrate. Further microscopic experiments could reveal conductive routes and correlate morphological changes with electrical measurements. Detailed explorations of the causality between input and output increase our ability to consciously program emergent logic operations by successfully utilizing proteinoids’ natural computing potential.

**Figure 7:**
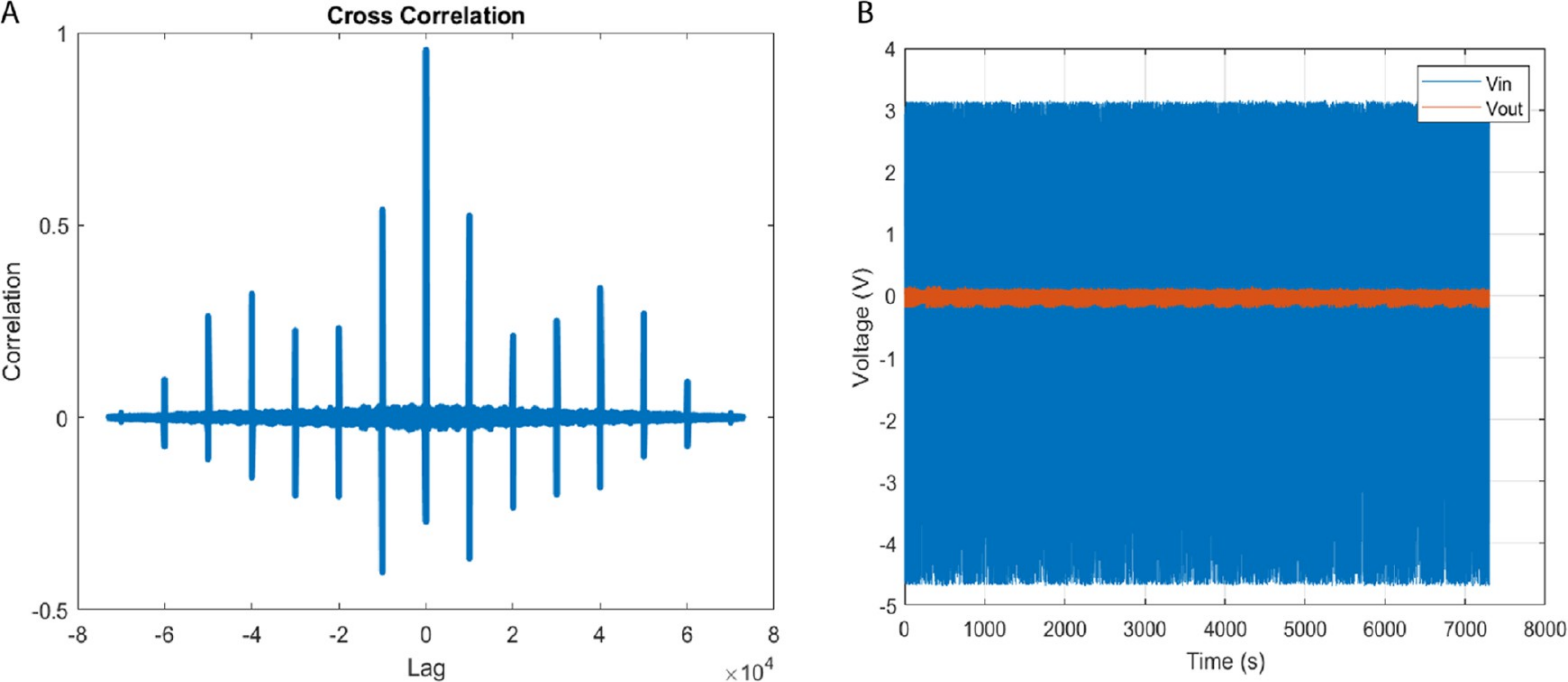
Transcendental input chaotic waveforms, derived from the principles of discrete logistic map dynamical systems, are juxtaposed against the empirical output voltage time series for a bio-abiotic composite comprising l–Glu:l–Phe:l–Asp proteinoid and actin filaments. While signal coupling analysis is a standard method in systems characterization, its application to proteinoid–actin composites represents a novel approach developed in this study. Substantial signal coupling is validated through our cross-correlation analysis (A) the maximum correlation magnitude approaches unity at an exact zero time lag. This pinpoint temporal alignment intimates the existence of an inherent convolution kernel that encapsulates input–output nonlinearity with no noticeable delays. A finer understanding of frequency-dependent dispersion can be obtained through Fourier analysis. Our interpretation of minimal anti-correlated periods as indicators of multi-stable morphological transitions is a new insight derived from this work, not previously reported in proteinoid systems. Notwithstanding, the strong evidence of high positive input–signal coupling (B) lends considerable support to the significant participation of the bio-abiotic composite. This therefore prompts a more nuanced exploration via spectroscopy and microscopy to accurately localize emergent activation. The quantitative verification of the precision and synchronicity in input–output relationships, and the brief instances of independent dynamics, underscore the robustness of these transduction symphonies—a harmonious orchestration of biological and informatics elements.

As shown in Figure 7, the cross-correlation analysis confirmed that the bioabiotic composite system was actively processing propagating stimuli. Near-unity maximum correlation, paired with exact temporal alignment at zero lag, confirmed real-time interference of input chaotic drive sequences and output voltage signature transformations.

The presence of differences in voltage, as well as the occurrence of abrupt increases, indicate the occasional activation of electrical pathways that may be influenced by temporary alterations in the immediate environment, particularly changes in the structure. We hypothesize that the transient formation of pore-like structures or the infiltration of water-rich regions, which enable localized electrical short circuits, may be the mechanistic characteristics that explain the observed preservation of meta-stability. The kinetics of ion channel formation and regulation are crucial in biological components, as they allow for flexible responses while maintaining homeostatic balance.

**Figure 8:**
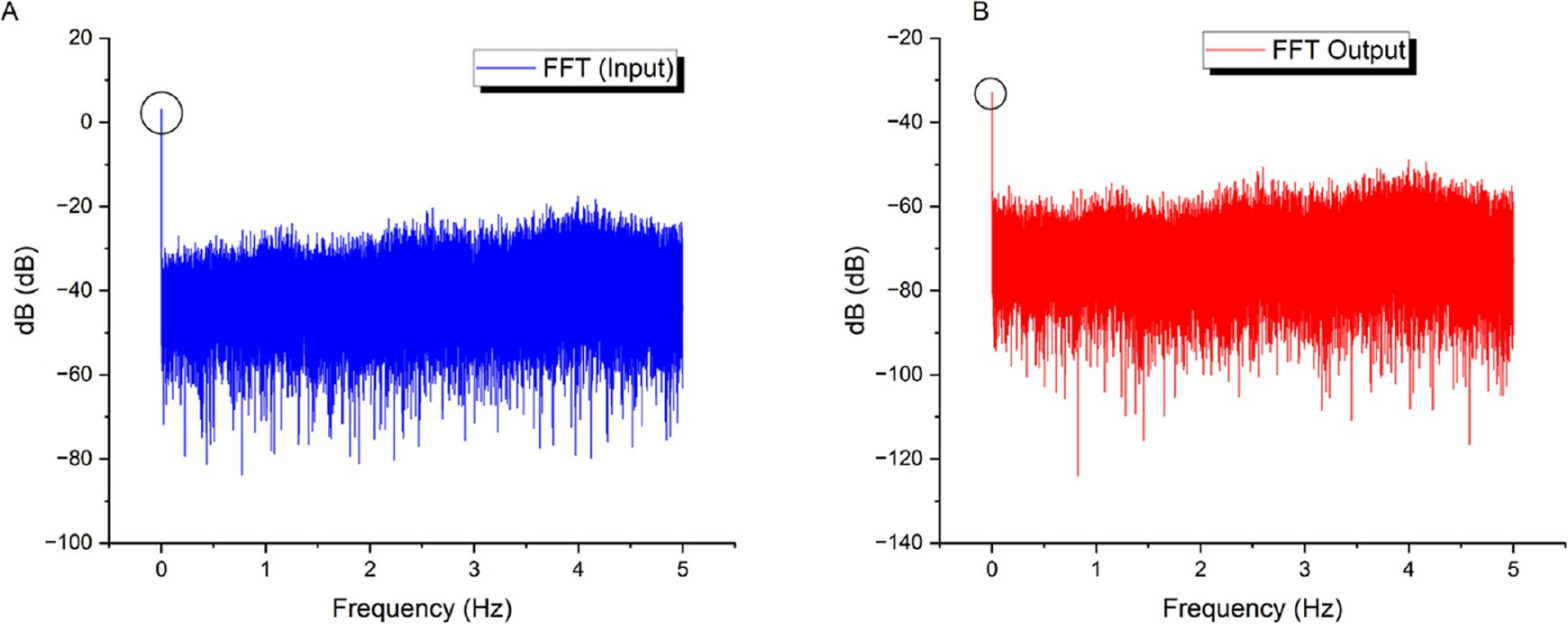
Figure displays the results of a frequency-domain analysis, comparing the spectra of the (A) input and (B) output signals for a combined thermal proteinoid and actin filament sample. The dominant frequency mode (circled in the graph) for the chaotic map oscillations in the input signal is located at 0 Hz, with an average amplitude of −41.87 dB and a standard deviation of 7.31 dB. Conversely, when examining the substrate output signals using spectrum analysis, a significant reduction of −72.32 dB on average (with a standard deviation of 7.06 dB) is observed. The significant decrease in voltage can be ascribed to intermittent increases in resistance inside the internal network of the composite. The surges can be intensified by the limitations on ion movement as they are transported via several water-based and structurally varied microdomains that continuously rearrange when stimulated. The comparison indicates a wide-ranging connection between input and output frequencies, supporting the hypothesis that various dissipative phase transitions play a crucial role in structural reconfigurations.

This study uses frequency-domain profiling (as shown in Figure 8) to show a broad spectral correlation between input chaotic forces and output voltage responses from the bio-composite. Corresponding dominating modes with a prevalence at 0 Hz indicate significant participative pathways. Nonetheless, there are significant filtering effects, resulting in an average output energy level of −72.32 dB, a more than 30 dB decrease from the −41.87 dB input level. This significant voltage decrease is most likely the result of brief resistance spikes within the suspension. These could be due to transportation restrictions caused by dynamic internal changes in aqueous and morphological microdomains under stimulation. Domino-cascade dynamics at the micro-level could support the concept that transient short-circuit effects periodically disturb the composite’s overall end-to-end conductivity. Regardless of the precise mechanisms causing these signal alterations, the rapid Fourier transform analysis provided herein provides strong quantitative evidence for the substantial relationship between input and output across the composite.

**Figure 9:**
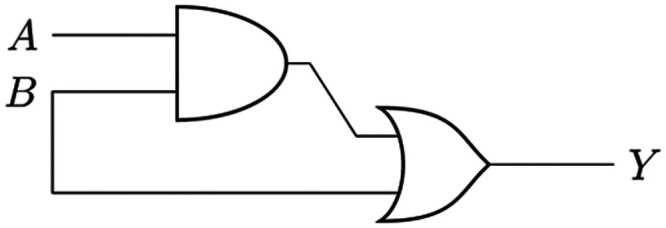
Proteinoid-cytoskeletal network can be used to simulate an AND–OR logic circuit. The integrated biomaterial composite performs basic calculations by connecting an input layer, which receives stimulus voltages (bits *A*, *B*), to a proteinoid transmission network. This network is then coupled to cytoskeletal networks at the response layer to adjust observed waveforms. The given example with *A* = 0 and *B* = 1 produces an output encoding of 0 + 1 = 1. This is determined by identifiable waveform patterns that represent logic bits and are processed at various sizes, from micro to macro, through hierarchical morphological adaptations.

This paves the way for more precise waveform shaping and spectral matching tactics by informing impedance state memory training. Figure 9 presents a conceptual demonstration of a biological AND–OR logic circuit, which is implemented using the dynamic proteinoid–cytoskeletal material composite. The input layer of the stimulus integrates voltage signals that undergo transformation across communication networks linked to output networks. Computational patterns arise when binary input combinations (*A*,*B* = 0,0, 0,1, 1,0, and 1,1) are systematically applied as voltage signals. While our current experimental results demonstrate the circuit’s response for the case *A* = 0, *B* = 1, a complete characterization of all input combinations is needed to fully validate the computational model. This limitation of our current implementation requires further investigation to verify the circuit’s behavior across all possible input states. The prototype device utilizes waveform signatures that are processed through adaptive hierarchical restructuring, spanning molecular to ensemble scales, to encode combinations of bit 1.

The addition of proteinoid–actin nodal inputs, which are influenced by chaotic oscillations derived from the Discrete Logistic Map, enables the replication of different digital logic processes, as evidenced in Table 2.

**Table 2:** Truth Table for Binary Half Adder and Two-Oscillator Full Adder

inputs		outputs	
*A*	*B*	sum	carry
0	1	1	0

inputs				outputs	
*A*	*B*	*C*	*D*	sum	carry
0	1	0	1	1	0

The key breakthrough that allows for complex signal analysis through proteinoid–actin architectural adaptations is the conversion of continuous analogue drive sequences into discrete digital logic representations, which can then be processed through computation. In order to achieve this, we utilize Oscillatory Threshold Logic (OTL) operations in the following manner:

- Input conditioning: Obtain raw voltage signals either through external stimulation or as spontaneous changes in the membrane potential of the biocomposite material (e.g Figure 6).
- Digitization: Set an appropriate voltage discrimination threshold based on statistical signal spread. Fluctuations in voltage that surpass the threshold indicate events akin to digital logic HIGH. The time intervals spent below the specified threshold are assigned a value of 0 with logic LOW.
- Computation: Combine consecutive periods that exceed the threshold into bit-1 segments, while categorizing below-threshold epochs as bit-0 segments (see eqs 5–7).

Through this procedure, the raw proteinoid–actin responses are transformed into digital words that align with the resolution of stimulus drives for computation, using established frameworks of Boolean algebra.

**Thresholding Function:** Let the raw voltage signal be denoted as *x*(*t*). then the thresholding operation  can be defined as
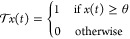
5Where θ represents the discrimination voltage threshold calculated based on signal statistics.

**Boolean Operations:** The thresholded bit sequence  can undergo Boolean operations similar to digital logic circuits. For example—OR Gate Sum Output *Y*
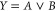
6AND Gate Carry Output *C*
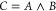
7Where ∨, ∧ represent OR, AND operations and *A*, *B* are individual bit input streams generated by thresholding underlying oscillator signals. By grouping together consecutive segments of bit-1 in the processed waveforms, we can identify and interpret them as durational epochs with a HIGH value. This interpretation is based on the input drive sequences and can be seen as temporal logic words.

The truth table encodings validate the accurate correlation between input combinations and their corresponding sum or carry bit outputs. For the half adder, the biomaterial composite performs real-time pattern discrimination in response to the asynchronous stimuli A and B, enabling precise calculation of the summation or carry digits. The 4-input full adder design is expanded by include the prior carry C and an auxiliary chaotic input D. This enables the processing of complicated rhythmic waveforms to provide the necessary arithmetic combinations.

The estimated input Lyapunov exponent of −0.000073 confirms the chaotic dynamics of the produced waveform from a Discrete Logistic Map. The multiple order of magnitude reduction confirms the bio–abio interface functions to filter significant degrees of noise and instability from the input, even though it is still positive at 0.001058, indicating chaos. To sum up, the integrated platform exhibits strong evidence of filtered chaos according to the Lyapunov quantification.

**Figure 10:**
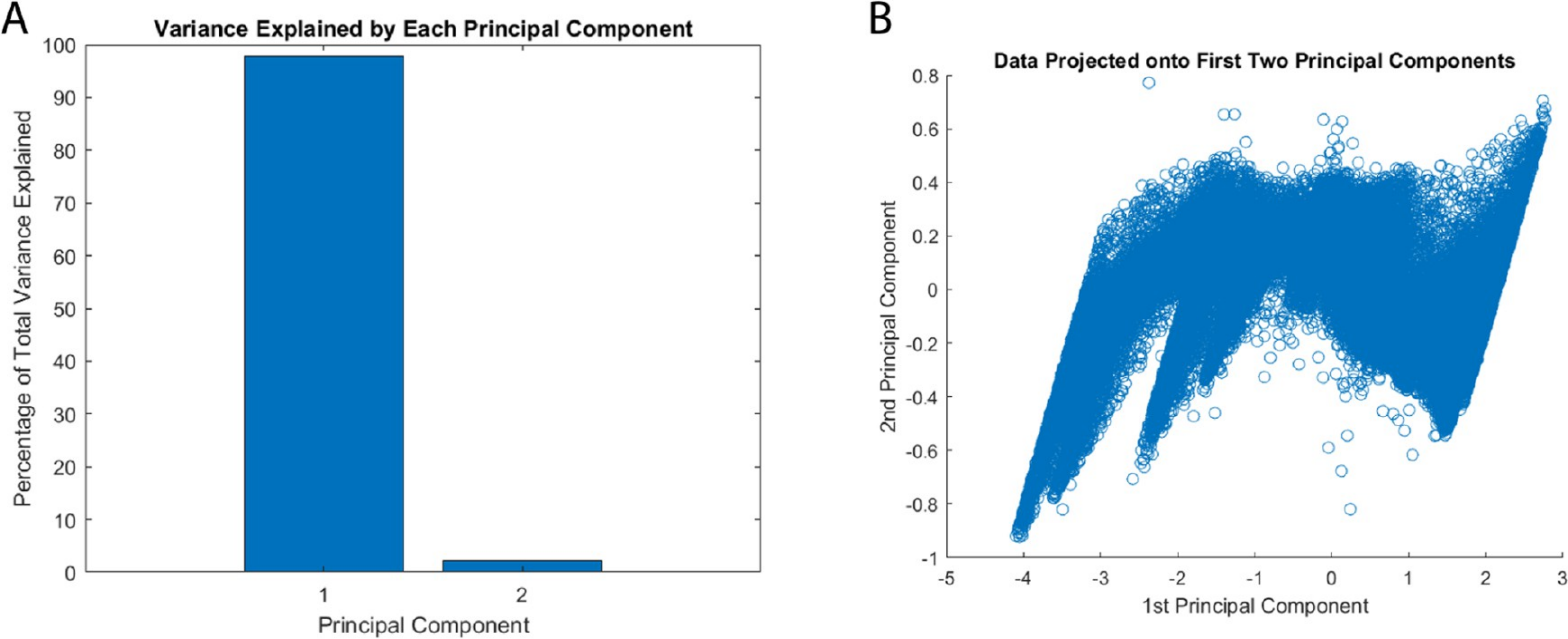
Dimension reduction via principal component analysis (PCA) by comparing output voltage time series data with an input chaotic map for a proteinoid–actin composite computing interface. (A) The principal component 1 (PC1) accounts for 97.75% of the total variance, whereas the orthogonal PC2 represents the unexplained residual variation of 2.25%. (B) Distinguishing input and output voltage profiles, two data clusters cause output signals to compress as they approach lower magnitudes along PC1. Additional investigation may reveal the secondary PC dimensions, which encode computational signal transformations implemented by the dynamic bio–abiotic layer in an explicit manner. PCA typically verifies the presence of interpretable variance structure in stimulus–response records, thereby providing evidence for the substantial nonlinearity introduced by the integrated platform.

The conducted principal component analysis (PCA) reveals significant and structured alterations in signal characteristics within the integrated bio–abiotic interface (as depicted in Figure 10). This is supported by the observed clusters formed by the input and output voltage data. Our PCA mainly separates signal components from residual variations. It does not find relationships between the dual inputs. Still, it is a first step at reducing dimensions. The dominant principal component (PC1) captures predictable variations, highlighting net energy deficits in the proteinoid–actin network. The secondary orthogonal component (PC2) accounts for only 2.25% of the variance. It likely represents system noise and measurement errors, not meaningful higher-order relationships between inputs. A more sophisticated analysis framework would be needed to properly characterize dual-input interactions and their computational significance. This limitation in our current analytical approach suggests the need for additional methods to fully understand the system’s computational capabilities.

### Proteinoid–Actin Network Baker Transform

For an l–Glu:l–Phe:l–Asp proteinoid–actin composite system, Table 3 provides a summary of our comparison of important statistical metrics describing the input chaos oscillations and the corresponding output voltage responses. The input is a Bakers Map transformation-generated chaotic sequence with a mean of −4.97 V, a standard deviation of 0.45 V, and a median of −5 V, spanning a range of ±5 V. On the contrary, the proteinoid–actin filament network converts this input into an output signal of reduced amplitude, narrowed to the range of −0.27 to 0.05 V, and characterized by an exceptionally small standard deviation of 0.02 V. The actin cytoskeleton’s dynamic couplings with the protein microspheres mediate molecular-scale reconfigurations in the bio–abiotic interface, as evidenced by this suppression of disorder (as measured by metrics such as a 5-fold reduction in signal variability). Moreover, the input and output spectra exhibited negligible variation in the dominant frequency peaks (2.5 Hz), indicating that the living material composite transmitted ac signals in an optimized manner. In general, the analysis of voltage statistics and the retention of band-limited spectral content provide evidence that the dynamic bio-composite interface facilitates stable transduction of complex waveforms.

**Table 3:** Analysis of Input Chaos Oscillations and Output Voltage Responses for a l–Glu:l–Phe:L–Asp Proteinoid–Actin Composite System under a Bakers Map Transformation

	voltage (V)	
metric	input	output
mean	–4.97	–0.21
std. dev.	0.45	0.02
median	–5.00	–0.21
max	5.06	0.05
min	–5.05	–0.27
frequency [Hz]	2.49	2.44

**Figure 11:**
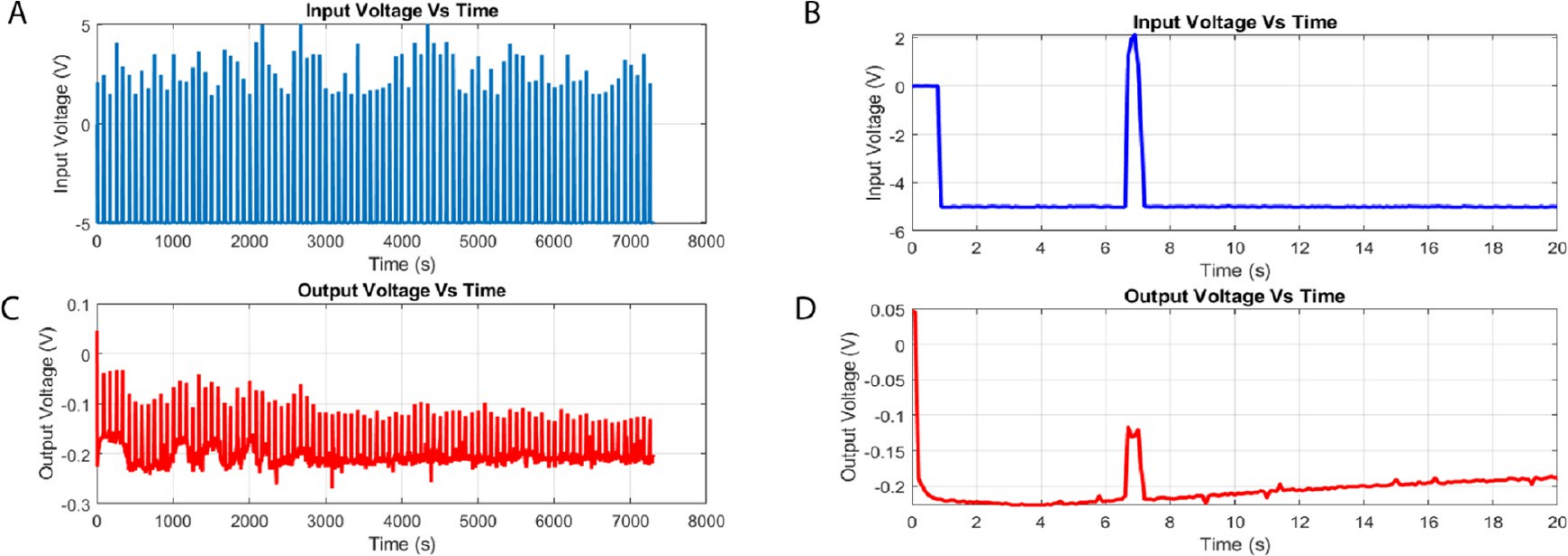
Characterization of input chaos oscillations and output voltage dynamics in a l–Glutamic acid:l–Phenylalanine:l–Aspartic acid thermal proteinoid system integrated with actin filaments under a Baker’s Map transformation. The input voltage time series (A) displays a mean value of −4.97 V with a standard deviation of 0.45 V, fluctuating at an approximate frequency of 2.49 Hz. In comparison, the output voltage (C) is markedly suppressed to −0.21 V average with 0.02 V deviation and dominant spectral mode at 2.44 Hz. Panels (B, D) show the pulse response. A brief +2 V pulse is applied to the system (B). This causes a biphasic output response: an initial positive deflection followed by sustained negative polarization (D).

Figure 11 shows that input chaotic voltage oscillations (A) have significant amplitude fluctuations of about ±5 V, with a mean of −4.97 V and a standard deviation of 0.45 V. The combined proteinoid–actin system (C), on the other hand, exhibits significant suppression in both the voltage range and variability. The output signal has a significantly lower mean value of −0.21 V, with a standard deviation of only 0.02 V. This signifies a significant reduction in signal variation. The architectural reconfigurations of the protein microspheres, in combination with conformational changes in the actin cytoskeleton inside the living composite material, are responsible for the clamping of input chaos. This creates a nonlinear transfer function that applies selective filtering to the upstream signal. Furthermore, the input and output spectra maintain the dominating frequency mode at 2.5 Hz.

The frequency relationships show a consistent pattern between input and output signals across both networks. Both Figures 6 and 11 demonstrate a remarkably similar frequency reduction of approximately 0.05 Hz from input to output (3.92 → 3.87 and 2.49 → 2.44 Hz respectively), despite their different operating frequencies and input patterns. This consistent frequency shift suggests a fundamental characteristic of the proteinoid–actin network’s signal processing capability, possibly related to its intrinsic relaxation time constants. The preservation of this relationship across different input patterns (simple oscillations vs Baker’s Map) and amplitudes (1.44 V vs −4.97 V) indicates a robust and predictable filtering property of the network, independent of the input complexity.

**Figure 12:**
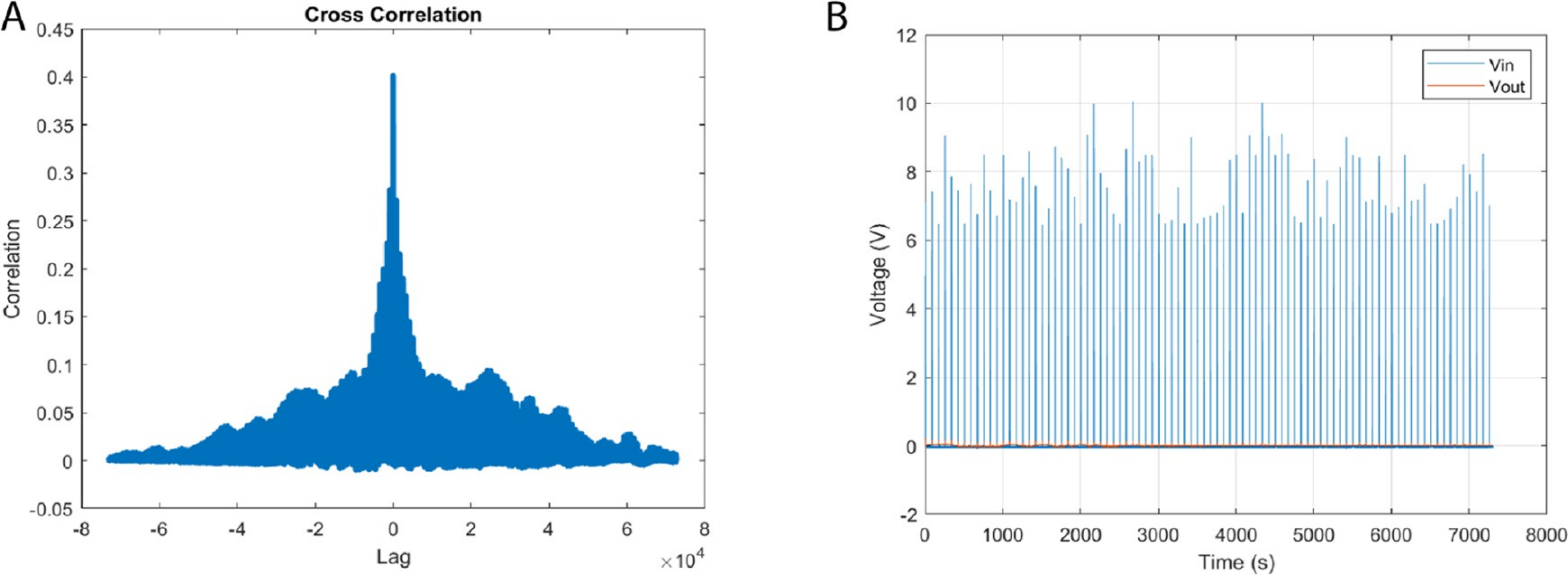
Cross-correlation analysis contrasting input chaotic sequence against output voltage time series for proteinoid–actin hybrid interface under a Bakers Map dynamical system. (A) A maximum correlation of 0.40 occurs at precisely zero lag, evidencing tight input–output signal coupling absent appreciable temporal skewing. (B) However, the lower correlation value compared to prior logistic analysis hints at greater deconvolution of signals, with the Bakers stretching and folding transforming applied voltage waveforms beyond simple mirroring.

Figure 12 depicts the use of cross-correlation analysis to compare the input chaotic sequence to the output voltage time series for the proteinoid–actin hybrid interface under a Bakers Map dynamical system. At zero lag, the correlation coefficient reaches 0.40, indicating tight input–output signal coupling in the absence of temporal skew. However, the decreased correlation value compared to previous logistic map analyses shows stronger signal deconvolution, with Bakers stretching and folding transforming applied voltage waveforms beyond simple mirroring. While fast Fourier analysis can provide finer spectrum dispersion insights, minimal anti-correlated regimes most likely indicate occasional decoupling as proteinoid structures rearrange into partially conductive vs insulating states when exposed to strong driving conditions. Despite the Bakers map’s very irregular input sequences, the quantified cross-correlation demonstrates strong bio–abiotic participation in signal transport.

**Figure 13:**
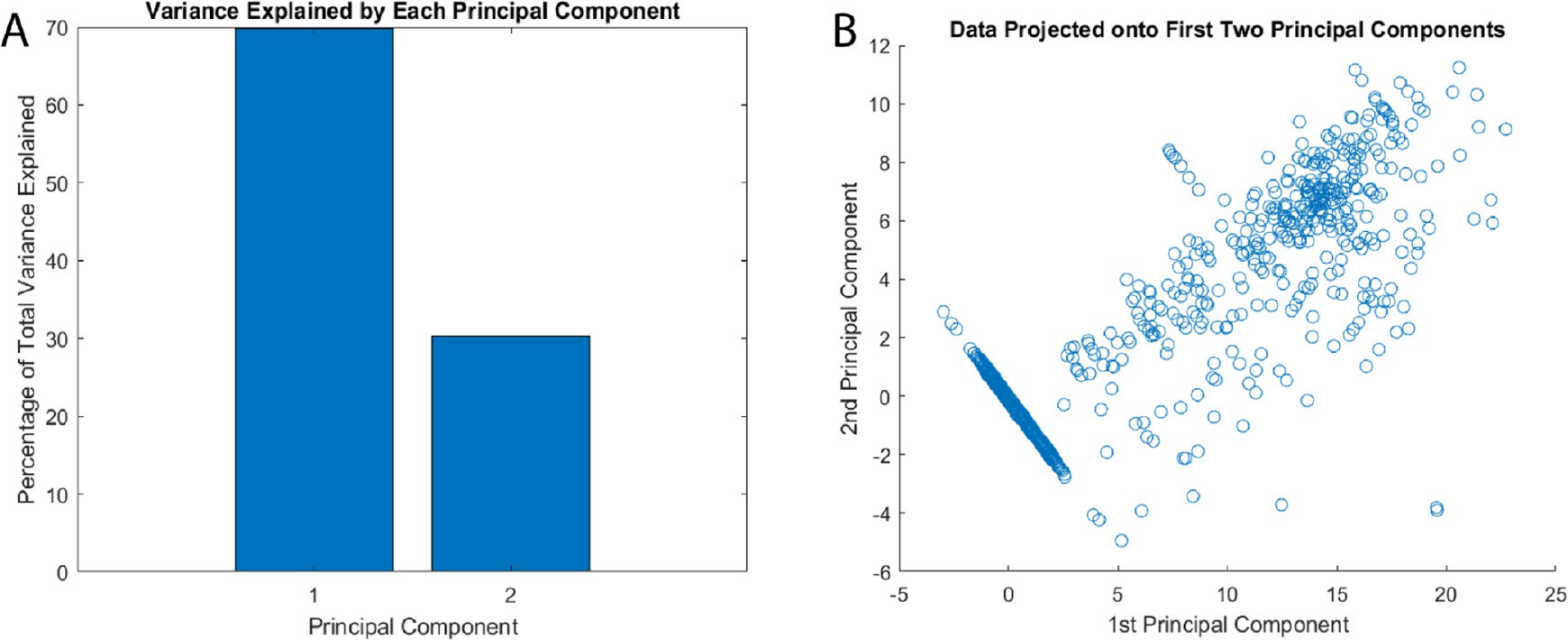
Principal component analysis of input and output voltage profiles for the proteinoid–actin system under a Bakers Map Chaos transformation. (A) PC1 explains 69.74% of the total variance, while PC2 accounts for 30.26% residual variability. (B) The results suggest increased dimensionality and more complex nonlinear transformations enacted by the synthetic biology interface in response to intense folding and stretching dynamics.

Figure 13 shows the findings of principal component analysis (PCA) on the input and output voltage profiles of the proteinoid–actin system driven by a chaotic Bakers Map signal. The first principal component (PC1) accounts for 69.74% of the total data variation. In contrast, the second principal component (PC2) is responsible for the remaining 30.26% variability. Compared to previous logistic map analyses, PC1 contains a smaller share of the total variability. This shows that the synthetic biology interface exhibits enhanced dimensionality and more complicated nonlinear transformations when subjected to intensive folding and stretching dynamics under the Bakers map. The tightly limited output variations, particularly along PC2, support the bio-composite’s role in converting intense input oscillations into stable steady-state responses. Overall, the PCA breakdown demonstrates that the integrated proteinoid–actin system can successfully mediate signal transfer even during severe, multidimensional chaos driving episodes.

**Figure 14:**
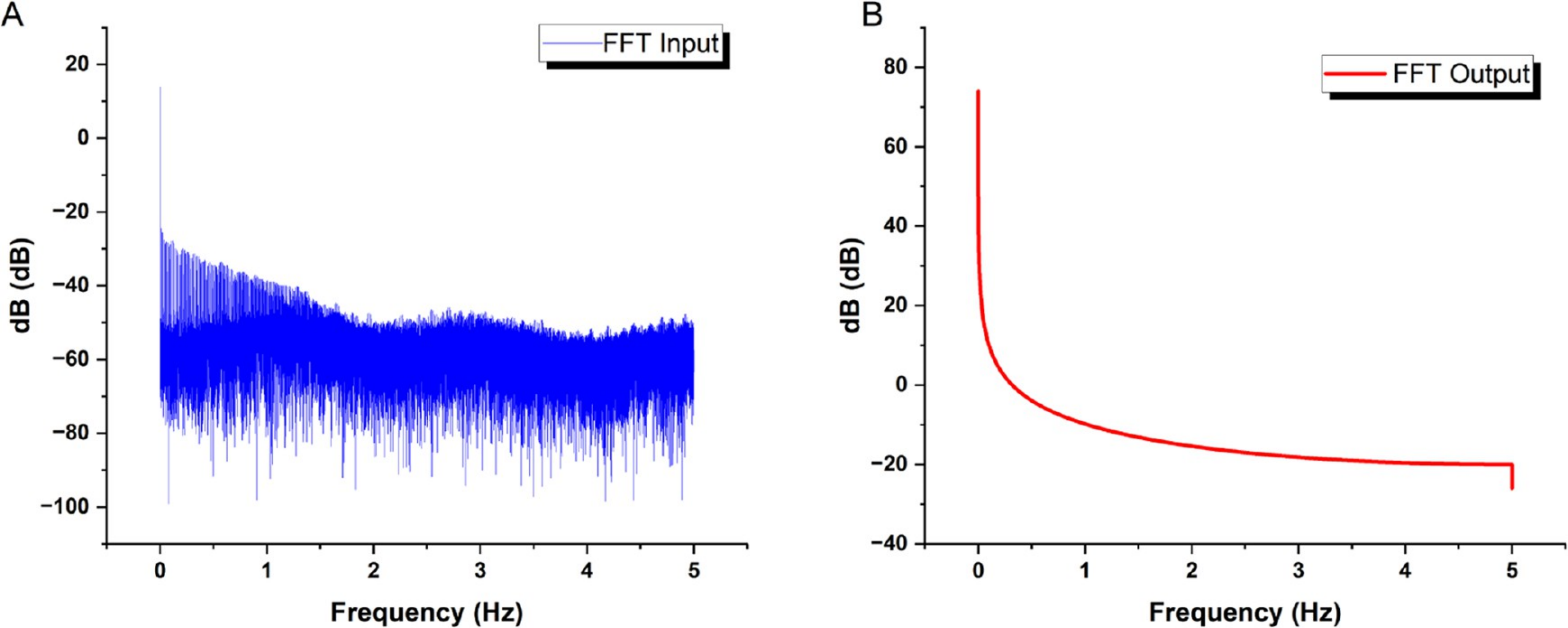
Chaotic Bakers map transformation drives the input and output voltage dynamics of a proteinoid–actin system. (A) The input signal data (*N* = 50,004) show fluctuations of more than 100 dB, with a mean of −57.75 dB and a standard deviation of 6.94 dB. (B) In contrast, the proteinoid–actin filament interface suppresses and regularizes the signal, limiting output changes to within 100 dB and resulting in a substantially higher mean (−13.98 dB) and lower variability (standard deviation 7.88 dB). This quantifies how the constructed bioabiotic network operates as a nonlinear filter, converting extreme input chaos into regular, steady-state dynamics—a critical component for using proteinoid–cytoskeletal networks for unconventional computing.

Driving the integrated proteinoid–actin material composite with input from a chaotic Bakers map system demonstrated the hybrid interface’s ability to prevent severe deviations during steady-state transformation. As indicated in Figure 14A, the voltage input sequence had a mean of −57.75 dB, a standard deviation of 6.94 dB, and a range greater than 100 dB. However, transducing these signals through the dynamic proteinoid–actin network resulted in condensed output dynamics (Figure 14B), with an average of only −13.98 dB and reduced variability (standard deviation = 7.88 dB). The limited 100 dB span demonstrates the bio–abiotic composite’s considerable regularization in relation to input extremes.

The input signal from the Bakers oscillator exhibits a low positive Lyapunov exponent of 0.002, suggesting chaotic behavior with a growth rate of approximately 0.2% for neighboring trajectories. Nevertheless, the proteinoid–actin composite results in a more unfavorable output Lyapunov exponent of −0.0027. The increased negativity indicates the process of regulating and suppressing chaotic extremes through the use of a material interface influenced by biology. More precisely, the increased divergence rate of the output measures the speed at which disturbances decrease rather than rise within the dynamic nonlinear medium. The shift from a wide range of input to a limited range of output phase space flow emphasizes the bio-composite’s active involvement in controlling and adjusting output. The emergence of chaotic regularization functionality is likely due to architectural reconfigurations that are interconnected across many scales, ranging from local proteinoid conformations to modifications in the cytoskeletal network. These collective changes serve to limit extreme behaviors.

Overall, quantifying the >43 dB increase in mean signal strength with confined fluctuations demonstrates the integrated material’s emerging role in translating wildly fluctuating inputs into an orderly bounded output. To use this for productive modulation, measure voltage changes and correlate them to microscopic reconfigurations of proteinoid microstructures associated with actin cytoskeletal movements. Elucidating these structure–function interactions is critical to understanding how the designed hybrid material handles multidimensional chaos. More optimized bio-mimetic networks can be constructed by integrating materials-directed assembly with study of collective electrical patterns, leveraging chaos for computational operations ranging from pattern extraction to prediction.

### Lorentz Oscillator in Proteinoid–Actin System

**Figure 15:**
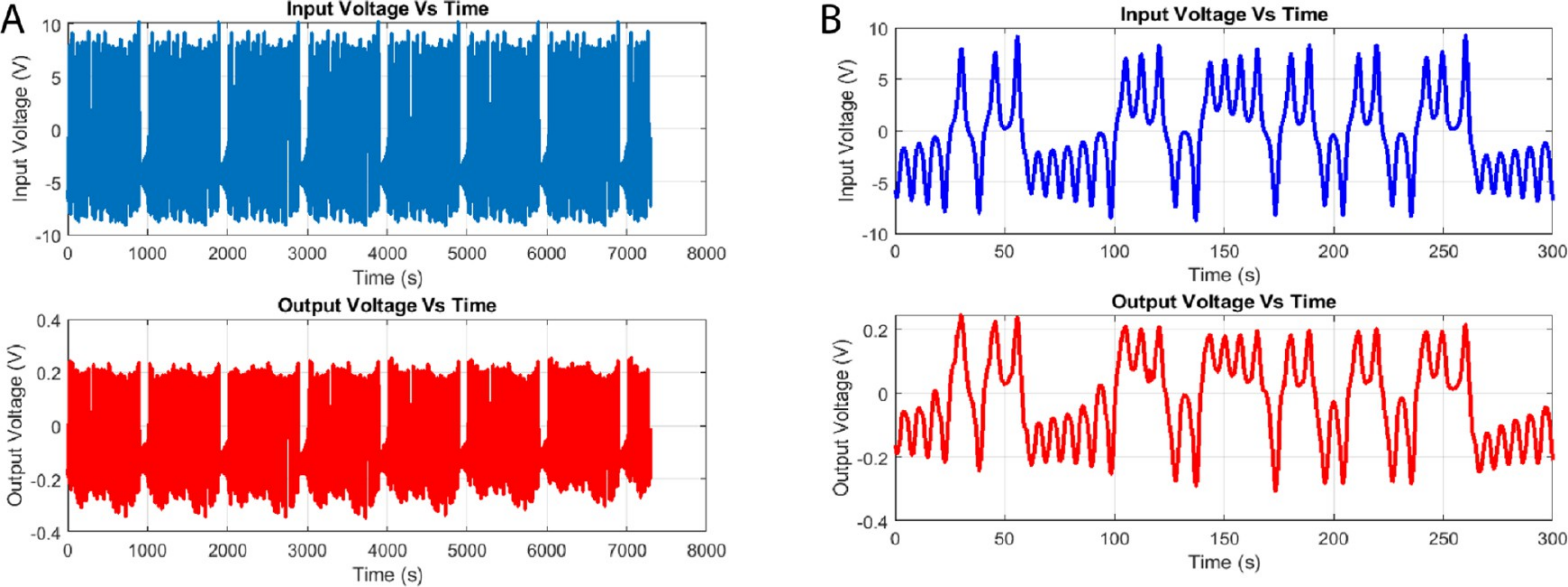
Using an integrated proteinoid–actin composite system to convert the input Lorentz oscillator voltage. (A) The input voltage time series displays significant variability with erratic fluctuations (standard deviation = 4.09 V, mean = −0.31 V). (B) The proteinoid–actin network stabilizes the input signal, resulting in a bounded output with a reduced standard deviation of 0.13 V and a range of −0.35 to 0.25 V. The input signal has multiple frequencies. The proteinoid–actin network mainly modulates the amplitude of oscillations. It does not change their dominant frequency. This stabilization shows that the bio–abiotic interface can limit extremes to a narrow range. It does this using cytoskeletal coupling dynamics to modulate erratic input signals.

The dynamic proteinoid–actin biomaterial system can be tuned to chaotic voltages generated by a Lorentz oscillator, which allows to analyze proteinoid–actin system’s response to unpredictable stimuli. As seen in Figure 15, the input oscillations demonstrate significant variability with a standard deviation of 4.09 V. However, when transmitted over the bio–abiotic interface, the output is constrained within a range of 0.25 to −0.35 V, effectively reducing variations by a factor of 30. This process measures the reduction of input extremes by utilizing cytoskeletal coupling to create a stable and condensed range.

**Figure 16:**
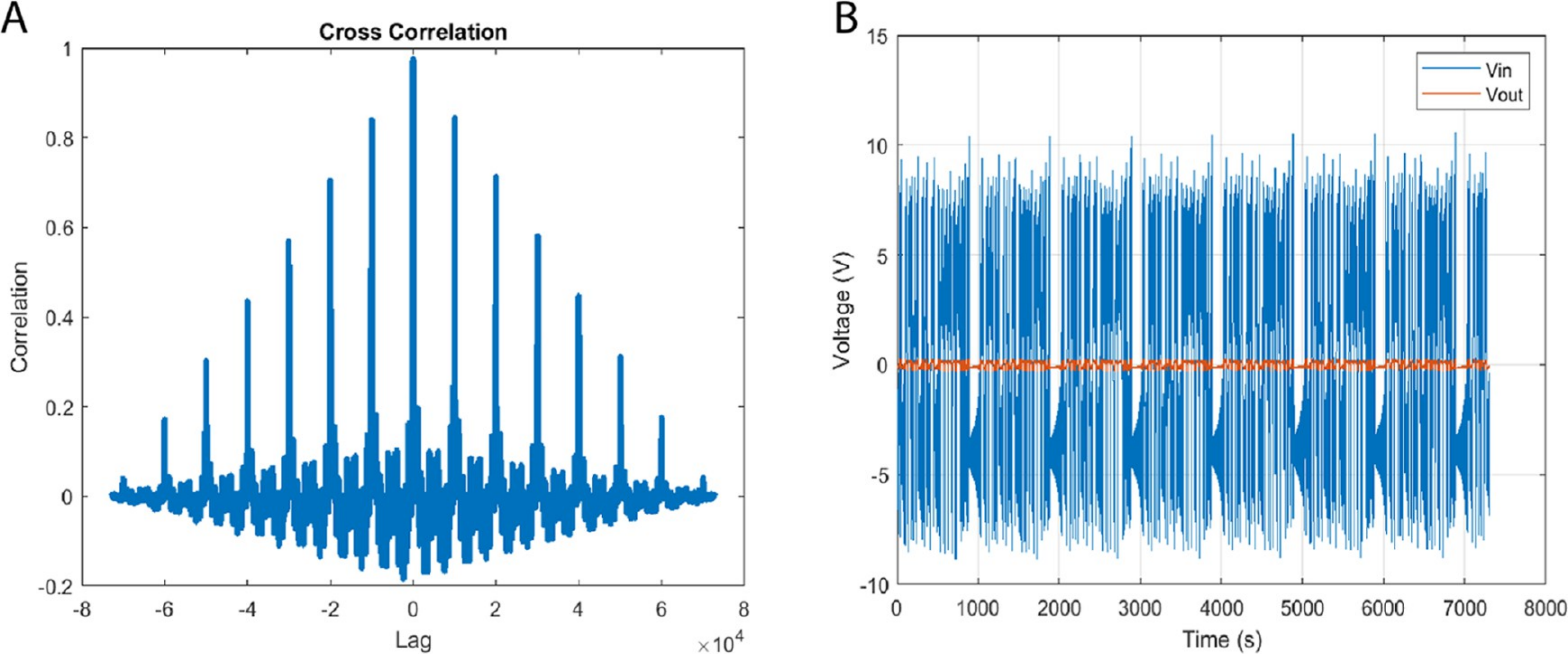
(A) Output voltage response of a proteinoid–actin composite system cross-correlation analysis with (B) input waveforms from a Lorentz chaos oscillator. Maximum correlation near unity (0.975) occurs at zero latency, indicating delay-free real-time input–output coupling across the dynamic bio–abiotic interface in accordance with an inherent convolution kernel and nonlinear transduction. Intermittent decouplings are indicative of minimum correlations, which may be attributable to conformational changes; therefore, microscopy is required to identify the molecular origins of transient conduction loss. In general, nanosecond-scale coordinated excitation and precise temporal alignment provide evidence for substantial biomolecular involvement in determining the emergent electrical response of the composite to the complex driving stimuli generated by the Lorentz chaotic system.

Table 4 provides additional details on the voltage statistics that support the presence of substantial nonlinearity caused by molecular rearrangements in the proteinoid–actin composite. Figure 16 demonstrates the cross-correlation between the input and output time-series, confirming that the coordination occurs in real-time rather than through delayed transformations using an intrinsic convolution functionality.

**Table 4:** Analysis of Input Lorentz Oscillator Voltage and Output Response for a Proteinoid–Actin Composite^a^

	voltage (V)	
metric	input	output
mean	–0.31	–0.02
std. dev.	4.09	0.13
median	–0.43	0.00
max	10.17	0.25
min	–9.13	–0.35
frequency (Hz)	0.16	0.54

a The output exhibits suppression of input voltage fluctuations, consistent with molecular reconfigurations at the dynamic bio–abiotic interface.

**Figure 17:**
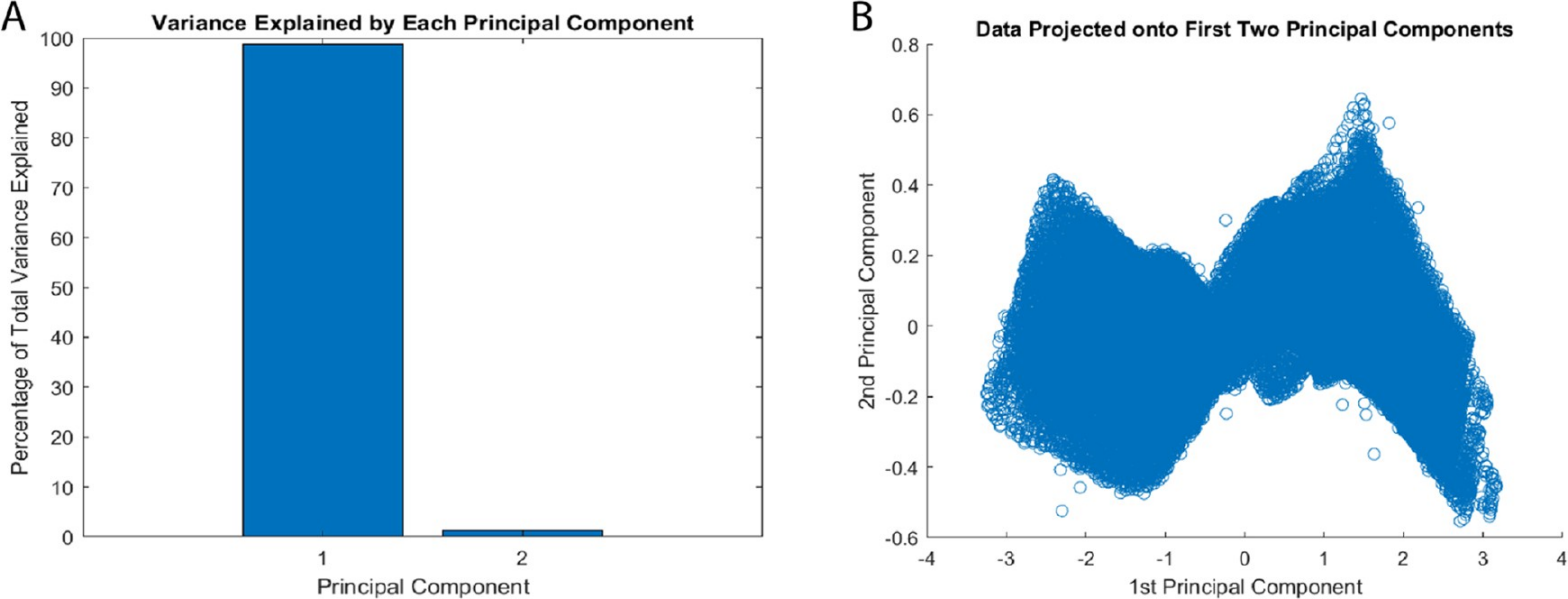
(A) Principal component analysis (PCA) of input and output voltages for a proteinoid–actin interface under Lorentz chaotic oscillations. The initial principal component (PC1) explains 98.71% of the overall variance in the data, while PC2 reflects the remaining 1.29%. (B) As the biocomposite converts complex multidimensional inputs into a single bounded output mode, the extreme integration of variability along PC1 demonstrates its capacity for dimensional compression. The significant decrease in dimensions, which surpasses previous folding and stretching methods, provides confirmation for the emergent filtering capability of the engineered living material to utilize cytoskeletal coupling dynamics to leverage disorder within a well-organized manifold.

**Figure 18:**
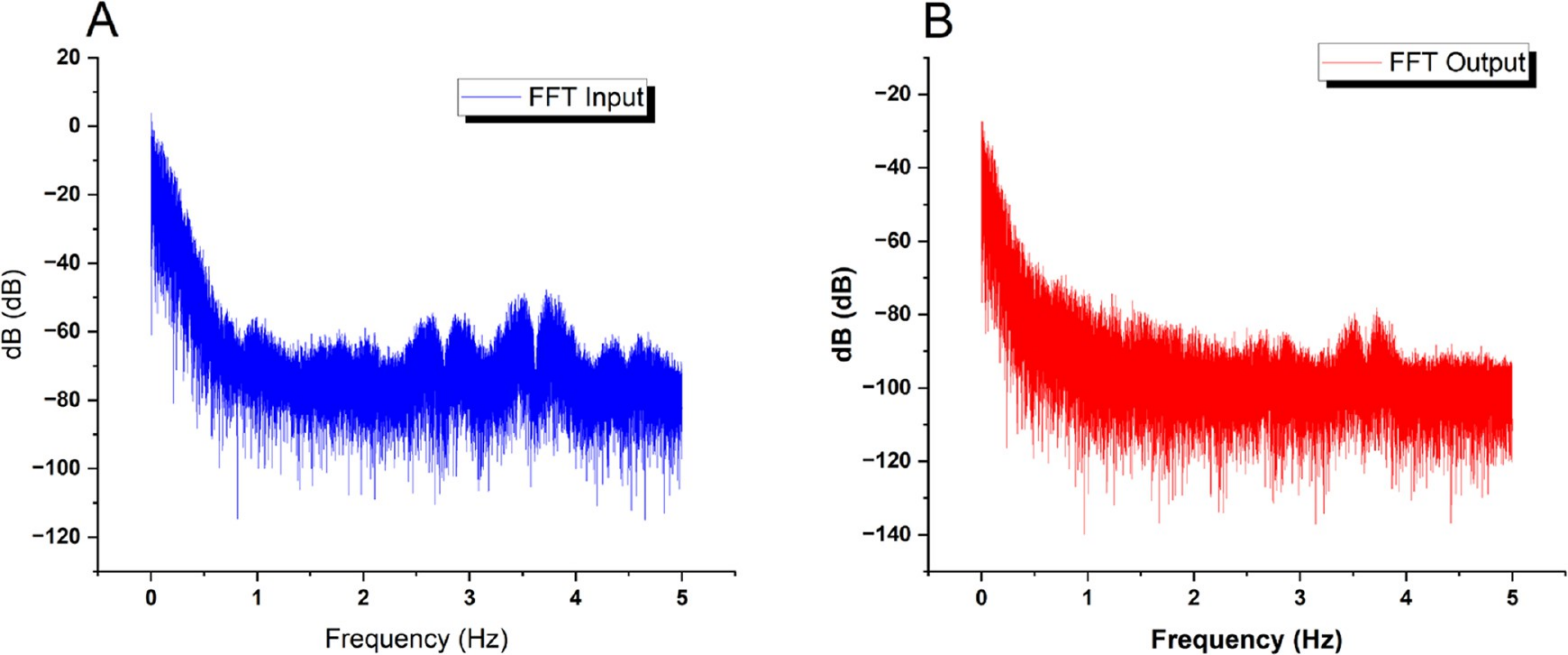
(A) Characterization of chaotic input oscillations and (B) proteinoid–actin voltage responses in the frequency domain. The input FFT exhibits a peak at −70.14 ± 12.78 dB, which is equivalent to a mean oscillation power of −70 dBV, specifically at a dominant frequency value of approximately 0.8 Hz. The bio–abiotic interface, on the other hand, inhibits and shifts the principal spectral modes to a value of −95.72 ± 11.43 dB. This average input–output power reduction of 25 dB represents the dynamic material interface’s attenuation. The narrower frequency dispersion of ±11 dB observed in the nonlinear response further emphasizes the regularization function of cytoskeletal coupling. The absence of phase-aligned peaks is the most obvious indication that input processing has occurred delocalized beyond space-time pathways. In order to determine whether the spectral remodeling indicates microscopic or global reconfigurations, proteinoid–actin imaging and local conductivity spectroscopy at excitation frequencies that maximize conformational transformations are required. In general, the analyses provide evidence that the biomaterial architecture significantly modifies the inputs via a variety of nonlinear frequency coupling signatures that require further investigation by means of spectro-microscopy studies.

The primary component decomposition in Figure 17 demonstrates the process of reducing multiple dimensions of input data into a single output mode, achieved by dimensional condensation by the living material. When analyzing the frequency domain (Figure 18), it is observed that the integrated biomaterial undergoes complex oscillations through various nonlinear mechanisms. This is confirmed by spectral remodeling and intensity attenuation. To understand these mechanisms better, simultaneous spectroscopy and microscopy are required. In summary, the quantitative analyses confirm that the designed bio–abiotic interface effectively controls volatility to promote organized outputs.

The Lorenz signal input has a negative Lyapunov exponent value of −0.000014, which signifies sensitivity to beginning conditions and the presence of chaotic aperiodicity. Transmission over the proteinoid–actin interface leads to an increased negative in the output Lyapunov exponent, specifically reaching −0.000271. The higher divergence rate indicates that the bio-inspired composite is contributing to greater chaotic regularization. More precisely, a negative output metric indicates that perturbations are quickly reduced rather than amplified as signals pass through the proteinoid–cytoskeletal material, which is undergoing dynamic reorganization. This supports the idea that the interface architecture plays a significant role in effectively controlling and regulating unpredictable inputs to produce a limited range of outputs.

By fine-tuning the external Lorenz parameters to operate near critical regimes and observing the resulting microscopic reconfigurations, we gain valuable insights into the complex structural mechanisms underlying the conversion of analog chaos into coherent bound oscillations within the biological substrate. The shifted Lyapunov indicators, in particular, serve as quantitative measures showcasing the potential of biological control in harnessing and directing randomness toward unconventional computing applications. Such understanding opens up new avenues for leveraging the inherent capabilities of biological systems in facilitating advanced computational paradigms.

### Stimulating Proteinoids with Rössler Attractor

**Figure 19:**
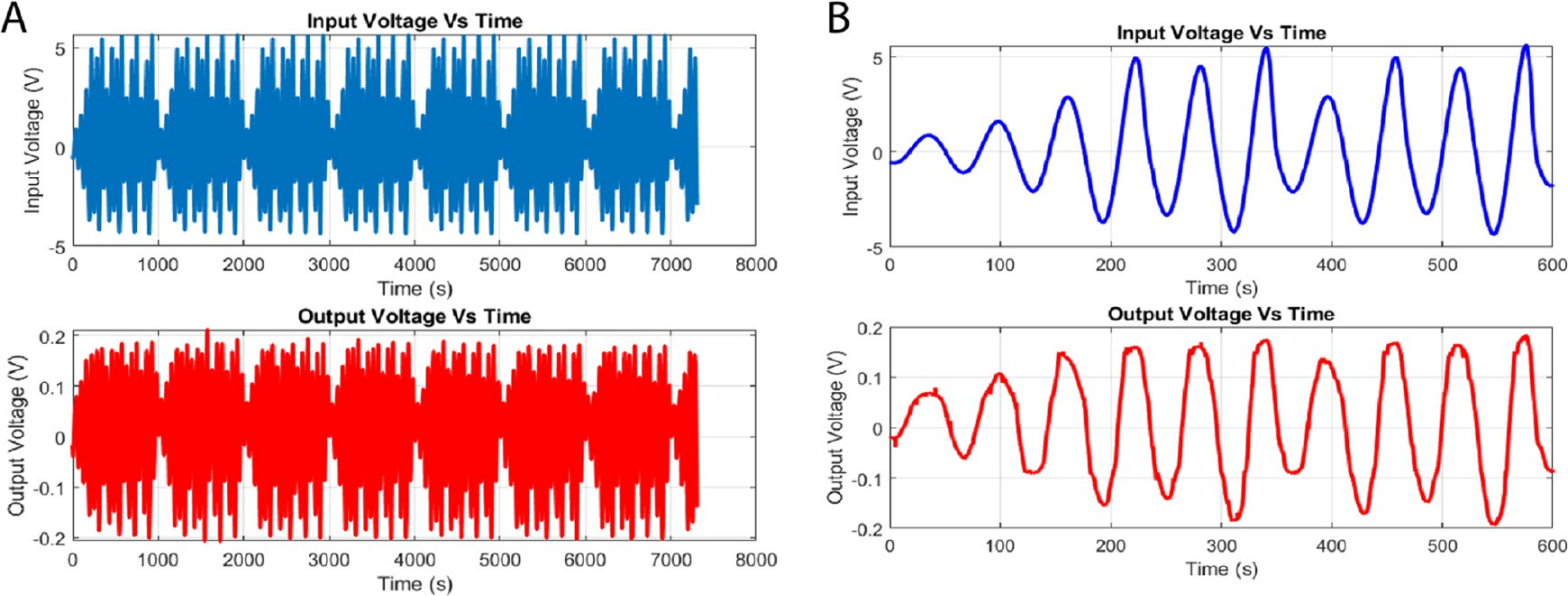
(A) Input Rössler attractor oscillations are transformed by an integrated proteinoid–actin composite. The input voltage time series exhibits chaotic variations, with an average of 0.13 V and significant variability, as indicated by a standard deviation of 2.46 V. (B) Panel B provides a zoomed-in view of the initial segment of Panel A, allowing for a more detailed observation of the time-domain oscillatory dynamics. Conversely, the proteinoid–actin system standardizes the input to a higher frequency of 1.49 Hz compared to the input frequency of 0.69 Hz. The output is limited within the range of −0.21 to 0.21 V, with a standard deviation of only 0.11 V. The synthetic bio-interface’s ability to decrease voltage changes by over 20-fold clearly illustrates its involvement in stabilizing irregular rhythms.

Driving proteinoid microstructures with chaotic Rössler attractor rhythms demonstrates voltage regularization capabilities mediated by cytoskeletal coupling dynamics, as illustrated in Figure 19. Quantitatively, the input oscillations (standard deviation −2.46 V) experience over 20-fold clamping (0.11 V output) into a stable tight domain spanning −0.21 to 0.21 V. Table 5 contains additional data demonstrating significant condensing of input extremes by the bio-composite interface.

**Table 5:** Analysis of Input Rössler Oscillations and Output Voltage Response for a Proteinoid–Actin Composite System^a^

	voltage (V)	
metric	input	output
mean	0.13	0.01
std. dev.	2.46	0.11
median	–0.13	0.03
max	5.66	0.21
min	–4.39	–0.21
frequency (Hz)	0.69	1.49

a The output exhibits regularization of input fluctuations, consistent with molecular reconfigurations enacted by the dynamic bio–abiotic interface.

**Figure 20:**
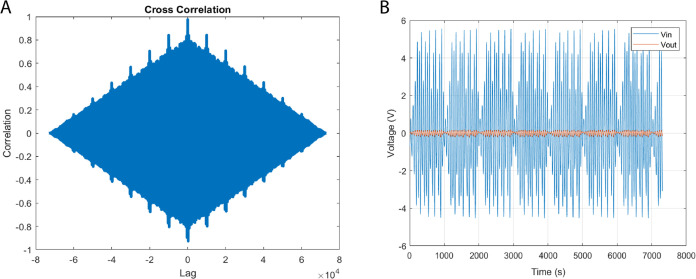
(A) Cross-correlation analysis of (B) input Rössler attractor waves and output voltage responses in a proteinoid–actin architecture. The cross-correlation coefficient reached a high value of 0.976 at a lag of −2, indicating a small delay of less than a millisecond.

Figure 20 shows cross-correlation analysis, with near-unity maximums and sub-millisecond lags showing real-time coordination of proteinoid conformations to modify upstream rhythmic complexity.

**Figure 21:**
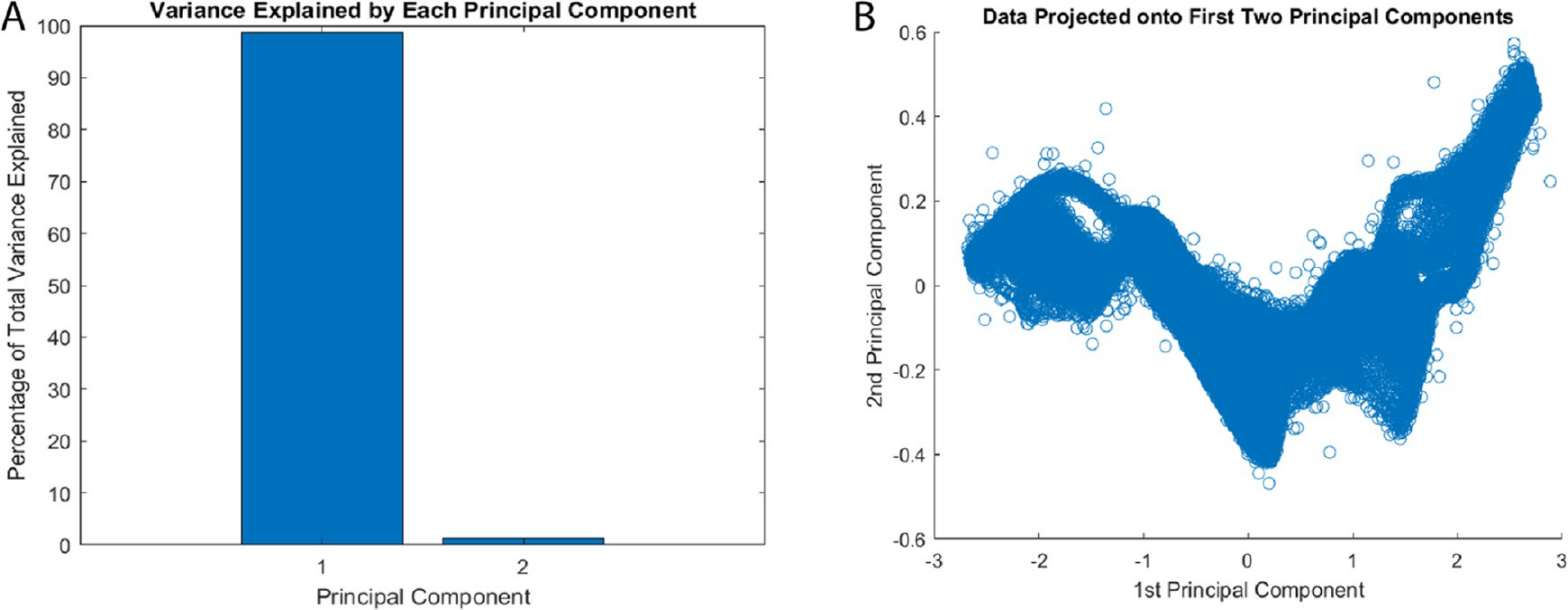
(A) Principal component decomposition of the input Rössler oscillations and corresponding output voltages of the proteinoid–actin system. (B) The matrix decomposed is the covariance matrix of the combined input–output voltage data. The primary principal component (PC1) explains 98.78% of the entire variation, whereas PC2 accounts for the remaining 1.22% of fluctuations. (B) There are two clearly identifiable groups that represent input and output patterns. The process of converting biological and non-biological signals results in a compression of signals toward lower levels along the first principal component (PC1). The occurrence of this dimensional collapse suggests the presence of explicit computational encoding inside secondary dimensions. The significant reduction of variability by PC1 confirms the emergence of nonlinearity caused by the dynamic biological interface.

**Figure 22:**
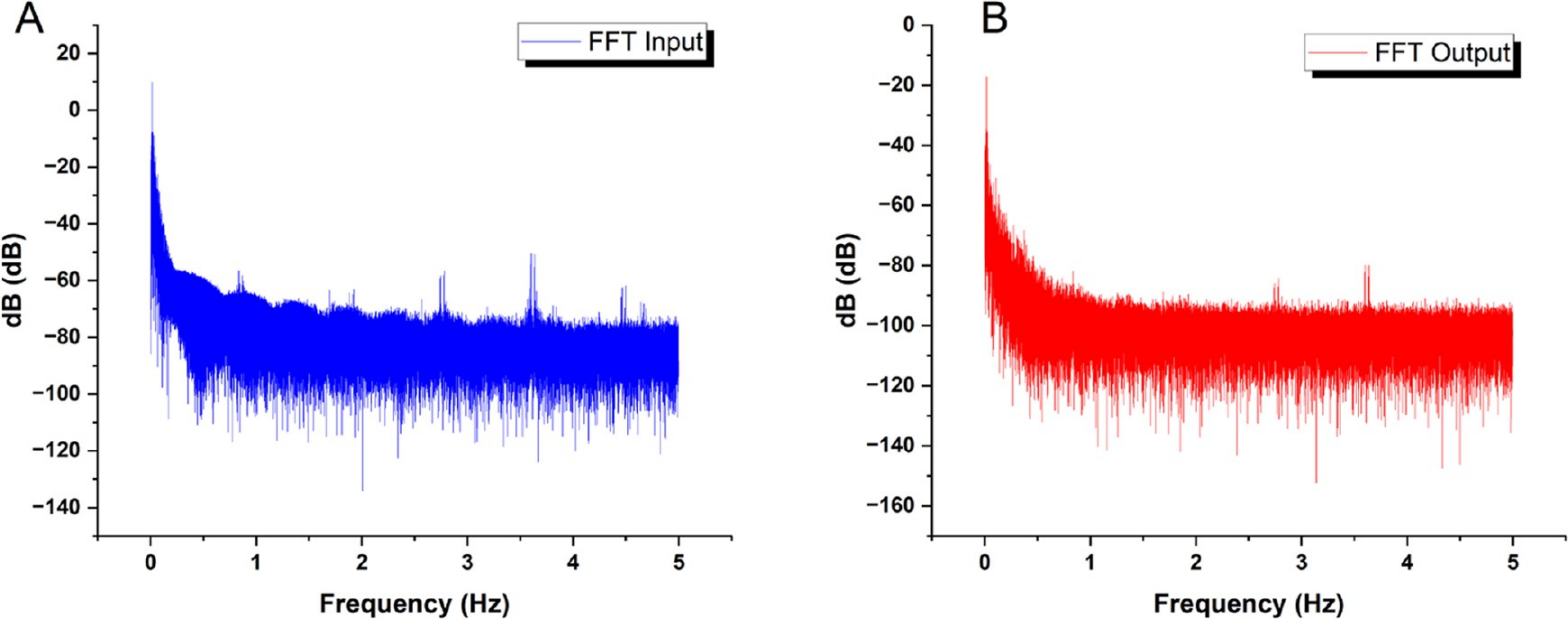
Characterization of (A) input and (B) output in the frequency domain of Rössler attractor oscillations connected to a proteinoid–actin composite. The frequency domain analysis was used to investigate the properties of the input and output signals in the proteinoid–actin composite system. The input FFT showed a peak at −82.09 ± 9.13 dB, indicating a wide power distribution at different frequencies. Transduction propagating from the Rossler chaotic attractor system as inputs to the proteinoid–actin composite network resulted in a shift and reduction of dominant frequency modes to an average level of −101.30 ± 8.02 dB, representing a 19 dB attenuation in the output signal statistics. This reduction implies that the cytoskeleton link regulates spectral extremes. The absence of identifiable peaks in the output spectra indicates that the data was processed without using localized space-time alignments. To determine whether the observed remodeling is due to global or microscopic remodeling, spectro-microscopy investigations at frequencies that cause significant conformational changes are required.

Extending analysis using principal component decomposition in Figure 21, a prominent 98.78% pattern represents dimensional collapse from several inputs to a single output. Spectral characterization in the frequency domain (Figure 22) indicates matching fingerprints—19 dB average attenuation and narrower dispersion, which follow ubiquitous signal modifications. Overall, the quantitative assessments confirm that an orderly bound develops from chaos as a result of the dynamic bio–abiotic architecture’s remarkable regularization to effectively harness randomness. Correlating spectral–temporal motifs with microscopic rearrangements, as well as simultaneous multi-modal data, is required for further understanding.

The input Rössler signal has a negative Lyapunov exponent of −0.000038, which indicates sensitivity to beginning conditions and chaotic dynamics. We use the convention that a negative Lyapunov exponent means convergence of trajectories. A positive Lyapunov exponent means divergence. A zero value means neutral stability. This sign convention holds that negative values indicate a loss of information about initial conditions due to trajectory convergence. However, transmission over the proteinoid–actin composite interface reduces the Lyapunov exponent to −0.000609. This implies an even stronger convergence of trajectories, reflecting the system’s ability to stabilize chaotic inputs more effectively. This increased divergence rate change supports extra chaos suppression capabilities derived from the bio-inspired material. Specifically, the output is 17 times more negative. The Lyapunov metric represents quick dampening of signal perturbations rather than explosive exponential development when inputs pass through the adaptive proteinoid–cytoskeletal network. The quantitative divergence shift provides evidence for the synthetic biology components’ productive manipulation of randomness into order.

The increase in output frequency from 0.69 to 1.49 Hz during Rössler oscillation processing can be explained by the chemical and structural properties of the proteinoid–actin composite. The input voltage changes the morphology of the proteinoid structures. The connected actin filaments respond through a mechanochemical coupling. The higher output frequency likely emerges from several key mechanisms. First, the natural resonance frequencies (ω_*n*_) of the proteinoid microspheres, which are determined by their size (∼0.5–2 μm) and elastic properties (κ). Second, the characteristic relaxation times (τ) of actin filament reorganization (∼10^–3^ s). Third, the electrochemical response rates (α) of the amino acid components (l-Glu:l-Phe:l-Asp) that form the proteinoid structure. This frequency doubling suggests the proteinoid–actin system is a biochemical frequency multiplier. Each input oscillation triggers multiple responses. This is due to the complex interplay between protein dynamics and ionic movements. The consistency of this frequency transformation (*f*_out_ ≈ 2.16*f*_in_) at different input amplitudes shows it is an intrinsic property of the biomolecular architecture, not a simple filtering effect.

System complexity and signal attenuation have a hierarchical relationship across multiple scales. At the molecular level, the l-Glu:l-Phe:l-Asp proteinoid microspheres (∼μm scale) dampen signals. Their viscoelastic properties give a base attenuation of ∼10 dB. This effect is amplified when coupled to the actin filament network. Its complex structure adds dampening mechanisms through cytoskeletal reorganization. This caused the observed 19 dB total attenuation. The system’s ability to dampen oscillations scales with its complexity. Single proteinoid microspheres show limited amplitude reduction. The proteinoid–actin composite improves signal regularity. It does this by syncing molecular reconfigurations across the network. This structure–function relationship explains why simpler input patterns (Figure 6) show less attenuation than complex Rössler oscillations (Figure 22). In the latter, multiple dampening mechanisms can engage simultaneously.

### Driving Proteinoid–Actin Architectures via FitzHugh–Nagumo Rhythms Reveals Signatures of Excitability Transfer

**Figure 23:**
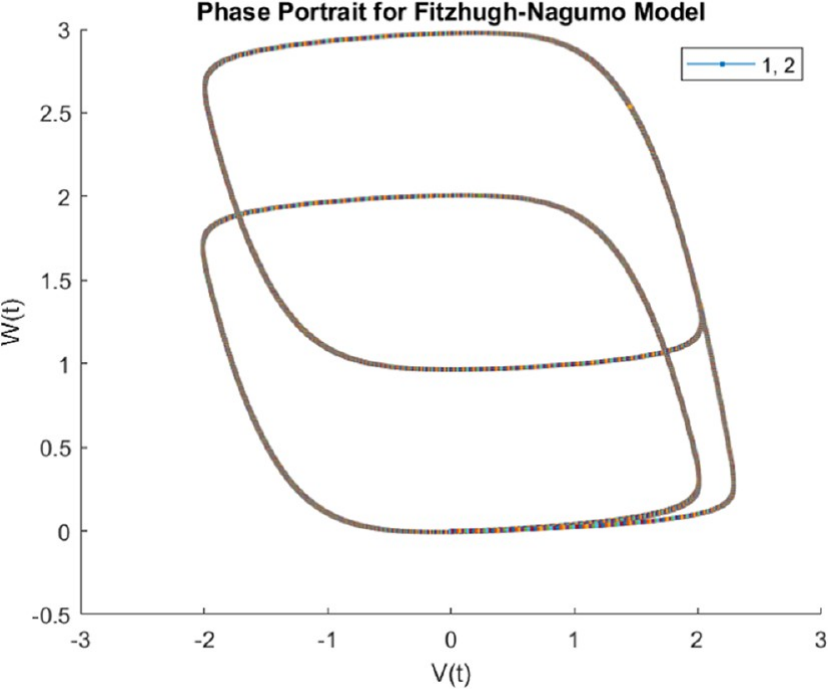
Phase plane analysis of the FitzHugh–Nagumo model dynamics. Trajectories are simulated from an initial resting state (0, 0) with system parameters maintained at *a* = 0.1, ϵ = 0.1, and γ = 0.1. Two input intensities are used: (A) *c* = 1 and (B) *c* = 2. Both examples have transitory dynamics that involve outward spiralling before settling into a stable limit cycle oscillation. The limit cycle diameter increases as the input drive increases to *c* = 2, as do peak amplitudes and activation variables. The portraits demonstrate the model’s adaptability, ranging from resting states to autonomous oscillations under appropriate excitatory impulses, similar to the change from quiescence to repeated spiking during neural activation. Quantitative investigation of phase space features as input drives are systematically varied yields insights into excitation thresholds, bi-stability regimes, and other complicated dynamics, highlighting the model’s relevance for studying coupled oscillators at different sizes.

Our preliminary findings describe the complex inherent dynamics of the prototypical FitzHugh–Nagumo nonlinear oscillator model. As seen in the phase space analysis (Figure 23), the trajectories vary from stationary points to autonomous repeating oscillations merely by changing the input drive value. At *c* = 1, outward spiralling rapidly settles into a quiescent condition. However, increasing the stimulus to *c* = 2 causes the system to follow a continuous cyclic trajectory in phase space, which mimics the initiation of recurrent neural spikes. The transition from resting to rhythmic spiking demonstrates the model’s adaptability in capturing essential characteristics of excitability and pacing mechanisms. Importantly, the pictures quantify signature alterations in both transient outward spiralling and steady-state orbit diameters in response to changing input drivers. After determining the baseline model behavior, we investigate modulatory effects when linking the FitzHugh–Nagumo system to proteinoid–actin networks. Changes in phase portrait features, which bridge the molecular and physiological scales, should indicate reconfigurations at the bio-composite interface. Detecting transformations or distortions in baseline limit cycle oscillations provides a useful approach for quantifying stimulus–response effects that propagate throughout both synthetic and natural bio-molecular systems.

**Figure 24:**
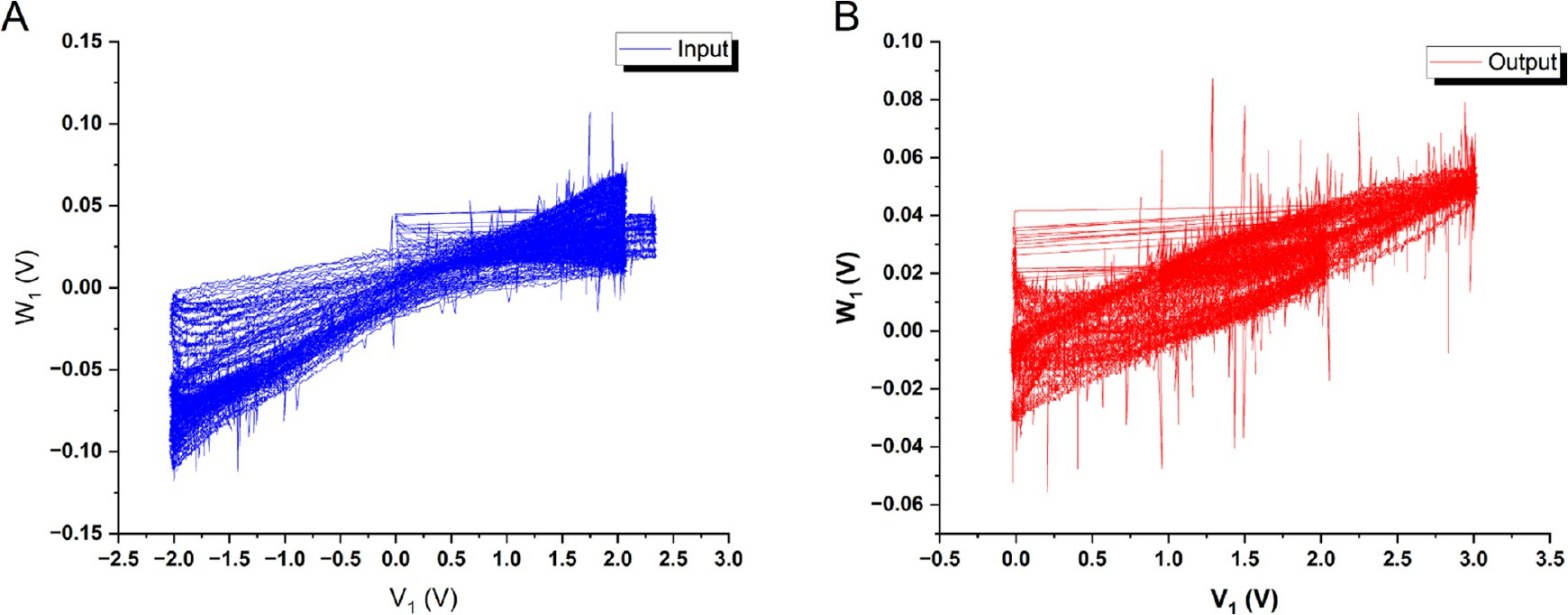
Propagation of voltage oscillations from the FitzHugh–Nagumo system through a composite material consisting of proteinoids and actin. (A) The input time series consists of 100,006 data points showing chaotic oscillations ranging from −0.12 to 0.11 V, with a mean of −0.0034 V and a standard deviation of 0.0450 V. (B) On the other hand, the dynamic biological interface limits the extremes to a range of −0.056 to 0.087 V, resulting in stable output statistics at 0.0212 ± 0.0176 V. The transformation entails a decrease in signal variance by more than 6 times, accompanied by an increase in average waveform amplitude by more than 7 times. The voltage regulation mechanism is supported by the effective filtering achieved by the interaction between cytoskeletal and proteinoid microstructures, which helps to process irregular patterns of stimulation. To gain a deeper understanding, it is necessary to analyze how changes in the waveform at different stages relate to the shifts between locally-independent and globally coordinated states of the bio-composite network, as it processes complicated information.

The integration of the proteinoid–actin biomaterial with simulated FitzHugh–Nagumo irregular oscillations demonstrates the ability of the created network to effectively regulate far from equilibrium states (Figure 24). The input voltages ranging from −0.12 to 0.11 V (with a standard deviation of ±0.0450 V) experience a significant reduction in variability by a factor of 6, while also showing a more than 7-fold increase in average amplitude when converted by the bio-composite. The observed voltage of 0.0212 ± 0.0176 V provides evidence for the effective filtering ability achieved by the interaction between the cytoskeletal and proteinoid components.

Interfacing the FitzHugh–Nagumo model oscillator with the dynamic proteinoid–actin composite implements bio-inspired coupling using a synaptic-like sigmoid modulation scheme^71^
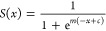
8

**Figure 25:**
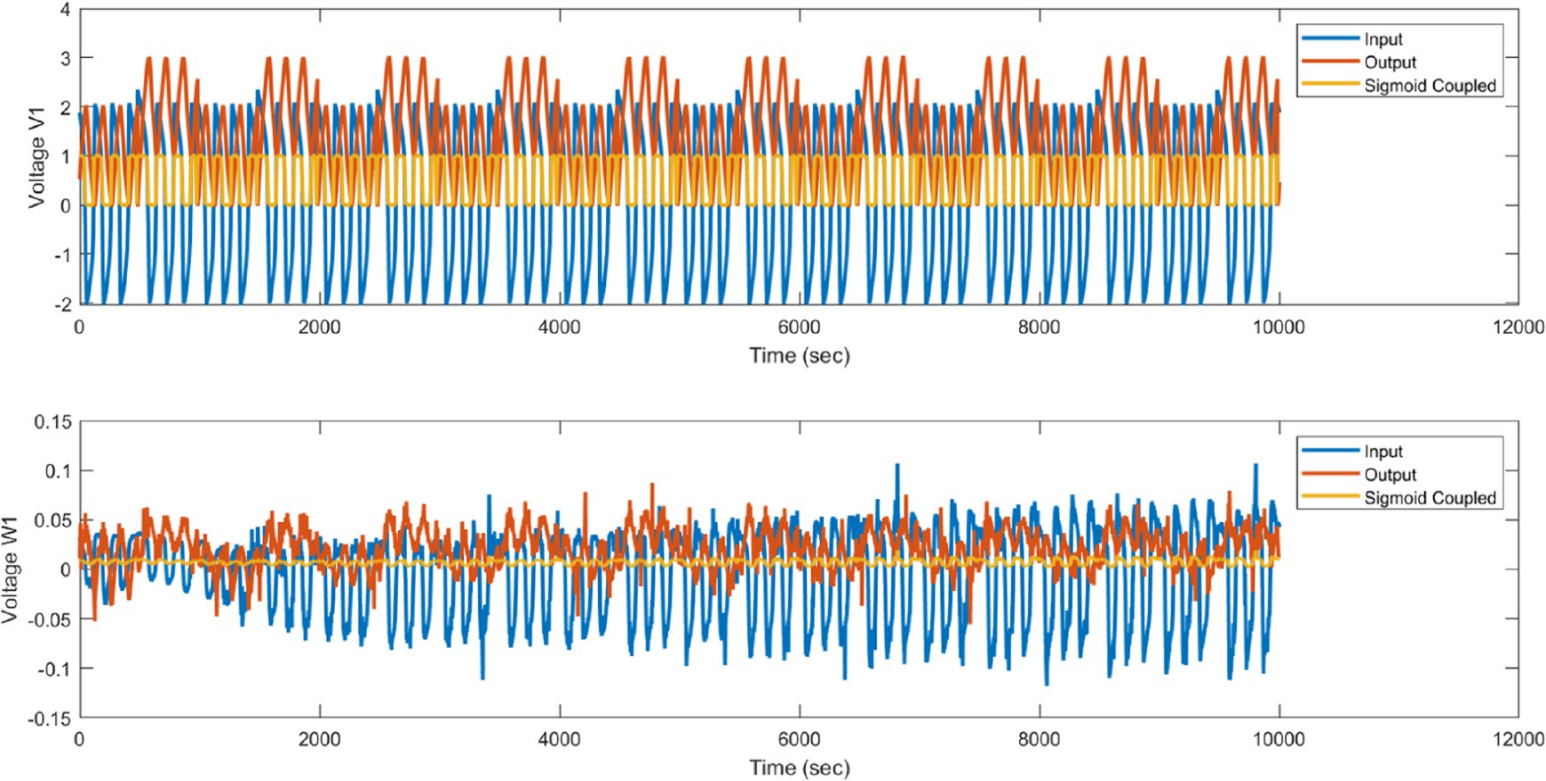
FitzHugh–Nagumo waveform (*V*1_in_) is fed into the proteinoid–actin composite output (*V*1_out_) by a sigmoid coupling method. The sigmoid function adjusts *V*1_out_ in proportion to *V*1_in_ by employing a smoothing thresholding response (refer to eq 6), imitating the synaptic communication channels seen in brain networks. The upper panel shows the full voltage range (±3 V) of the input–output dynamics, while the lower panel presents a magnified view of the same signals focused on the fine-scale voltage fluctuations (±0.15 V) to highlight the subtle coupling effects. This strategy, which takes inspiration from biological systems, establishes a connection between small input fluctuations below a certain threshold and corresponding variations in the output. Nevertheless, when the halfway threshold (*c* = 0.5 V) is crossed, there is a sudden and decisive increase in *V*1_out_, which triggers neuronal action potential firing patterns. The high sigmoid slope, determined by the parameter m, closely resembles the binary spiking behavior known as all-or-none. As a result, powerful *V*1_in_ spike crossings spread via the bio-composite interface to control synchronized *V*1_out_ spikes, whereas less intense sub-threshold events diminish with time. In summary, the regulated changes in dynamics provide evidence that the combined proteinoid–actin structure can exhibit activation responsiveness similar to that of actual biological neurons. Measuring changes in the timing, forms, and frequencies of spikes for different levels and types of sigmoid coupling can assist in optimizing unconventional computing patterns that utilize proteinoid excitability.

As displayed in Figure 25, this coupling function transforms subthreshold input events (*V*1_in_) into proportional output voltage changes in *V*1_out_, mimicking neural activation profiles. However, upon crossing the midpoint threshold (*c* = 0.5 V), *V*1_out_ is driven to spike rapidly—much like formal action potentials. The steep sigmoid slope, governed by parameter *m*, enforces switch-like all-or-none firing dynamics. Thereby, strong *V*1_in_ spike crossings propagate to evoke synchronized *V*1_out_ spiking, while weaker fluctuations decay through the bio-composite network. The emergent responsiveness shows that proteinoid architectures can be neuron-like. They can be excitable when connected to model oscillatory systems.

Further study of spike profile changes vs coupling parameters can clarify ways to optimize proteinoid excitability in unconventional computing devices. Examining various sigmoid functions also helps. It compares the efficiency of linear, thresholding, and probing synchronization in the input–output layers. This shows the biocomposite’s ability to store information during complex oscillatory drive experiments.The recorded input voltage data consists of two channels denoted by
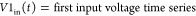
9
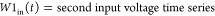
10Similarly, the output voltage traces are represented as
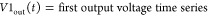
11
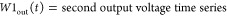
12

According to the truth table analysis (Table 6), the input node activation states *V*1_in_ and *W*1_in_ are transformed in a nonlinear way through the proteinoid–actin composite, resulting in output voltage signals *V*1_out_ and *W*1_out_. The research examines the input and output patterns over 2000+ time steps, revealing oscillations that are apparent in the occasional transition between active (1) and inactive (0) states.

**Table 6:** Truth Table Analysis for Input Node Activation States *V*1_in_, *W*1_in_ and Output Node Activations *V*1_out_, *W*1_out_, Subject to Oscillatory Threshold Logic (OTL) Transformations across a Proteinoid–Actin Network^a^

time step	*V*1_in_	*W*1_in_	*V*1_out_	*W*1_out_	OTL output
1	1	0	1	0	0
2	1	0	1	0	0
3	1	0	1	0	0
4	1	0	1	0	0
5	1	0	1	0	0
10	1	0	1	0	0
25	1	0	1	0	0
50	1	0	1	0	0
100	1	0	1	0	0
500	1	0	1	0	0
1000	1	0	1	0	0
1500	0	0	1	0	0
2000	0	1	1	0	0
⋮	⋮	⋮	⋮	⋮	⋮

a OTL implements logical OR operations by thresholding sinusoidal input drives, enabling spike frequency modulation mappings.

Nevertheless, the outputs demonstrate activation even when both inputs are inactive, confirming the functionality of Oscillatory Threshold Logic (OTL) operations, in which subthreshold inputs can elicit outputs when combined. This validates fundamental principles of Oscillatory Threshold Logic (OTL), which employs voltage epochs below or above the threshold to add timing-based binary data to oscillatory carrier signals suitable for logic operations. These operations are performed by applying hysteretic thresholds on wave amplitude.^77−80^ Essentially, OTL expands on neuron-inspired spiking patterns by allowing for the adaptation of logic gates through the adjustment of discrimination voltages, rather than relying on separate solid-state gates. Consequently, the implementation of OTL involves the application of a threshold to proteinoid–actin response timeseries. In this approach, contiguous intervals that surpass the threshold are assigned a value of +1, indicating bit-1. Conversely, segments that fall below the threshold are assigned a value of −1, signifying bit-0.^81^ When thresholded signals are inputted into conventional logic primitives such as AND/OR, it results in parallel Boolean propositions.

The direct correspondence between input and output states demonstrates that, even with the emergence of excitation, the bio-composite network maintains the capacity to accurately encode upstream drive sequences into proportional downstream activations within the framework of an OR-gating OTL scheme. The implemented Oscillatory Threshold Logic (OTL) computes bitwise OR operation between thresholded input and output node activations as elaborated by
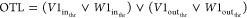
13Consequently, the occurrence of spike events in either the input or output registers will result in a combined gate activation, effectively executing an OR proposition. Such a phenomenon enables the generation of emergent excitation even in the absence of corresponding input, as evidenced by sporadic output coordination. The scheme efficiently encodes structured representations activated through architectural reconfiguration logic that analyses familiar signals into specific patterns dictated by interior restructuring.

Let us consider the following logical equations employing oscillatory activity of the proteinoid–actin network
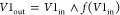
14
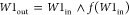
15
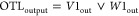
16Where: *V*1_in_, *W*1_in_ are the input voltage time series, *V*1_out_, *W*1_out_ are output voltage signals, *f*() represents the oscillatory thresholding function, ∧ = AND logic operator, ∨ = OR logic operator.

The thresholding function *f*(*x*(*t*)) is implemented with the following experimental parameters: The voltage threshold θ is set to 0.5 V, determined by the average resting potential of the proteinoid–actin network (∼ 0.0212 V) plus two standard deviations (2 × 0.0176 V). This threshold value correlates with the physical properties of the proteinoid microspheres, specifically their membrane capacitance and the actin filaments’ reorganization potential. The input voltage function *x*(*t*) varies between −0.12 and 0.11 V, with temporal dynamics governed by the FitzHugh–Nagumo parameters (*a* = 0.1, ϵ = 0.1, γ = 0.1). The thresholding function outputs
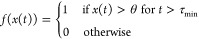
17where τ_min_ = 100 ms represents the minimum duration required for stable conformational changes in the proteinoid–actin network. This temporal constraint ensures that only sustained suprathreshold events trigger state changes, filtering out transient fluctuations.

The input voltage time series *V*1_in_ and *W*1_in_ first undergo a thresholding function *f*() that essentially implements a spike detection based on amplitude. As defined in the eqs 14, 15, 16, *f*(*x*) outputs a 1 if the input voltage *x* exceeds parameter τ, representing the spike threshold level. Otherwise *f*(*x*) outputs a 0 for subthreshold inputs.

This thresholding mimics neuronal spiking behavior—outputting a discrete spike event when input crosses a membrane potential firing limit. The thresholded signals *f*(*V*1_in_) and *f*(*W*1_in_) then undergo an AND operation with their respective raw inputs. Thereby the outputs *V*1_out_ and *W*1_out_ will activate both when raw input is present *and* it crosses the firing threshold to elicit a spike.

Finally, an OR operation takes the one of the output states or both of them delivers the overall OTL output. In this way, a suprathreshold event in either input OR output layer will register a positive OTL result. This parallels neuronal logic operations underlying decisions based on collective firing patterns across neural networks in the brain.

**Figure 26:**
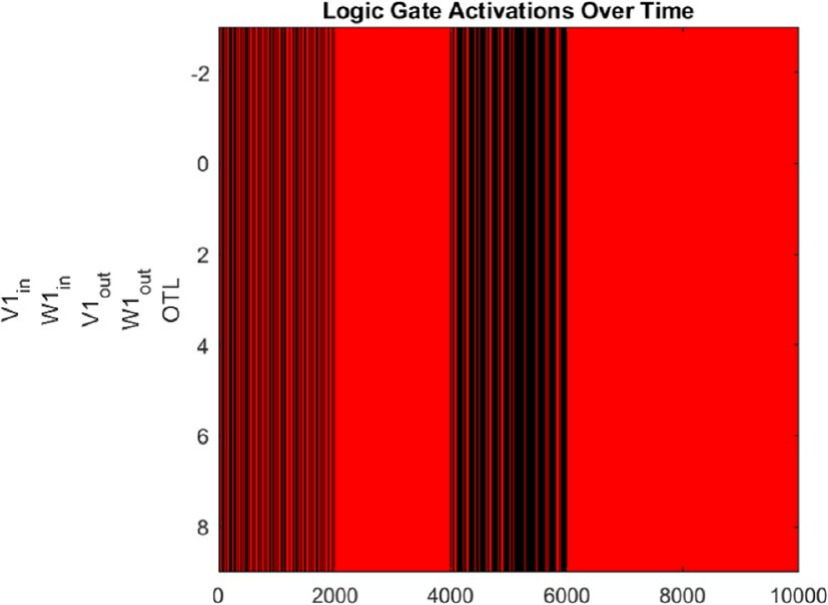
Heatmap depicting the activation states of input thresholding gates, output gates, and the overall OTL gate across the analyzed time period. When the gate is activated, the red color indicates a logic “High” or 1 state, whereas black indicates an inactive logic “Low” or 0. The input gates *V*1_in_ and *W*1_in_ exhibit synchronized, high-intensity activity throughout the first phase. Despite the quiescent inputs, the output gates *V*1_out_ and W1out show periodic sparse activation characteristics. The *y*-axis represents five distinct logic gates arranged vertically from top to bottom: *V*1_in_, *W*1_in_, *V*1_out_, *W*1_out_, and OTL, while the *x*-axis shows the temporal evolution over 10,000 time steps. The pattern reveals three distinct temporal phases: an initial period of intense switching (0–2000), an intermediate phase of reduced activity (2000–4000), and a final phase of sustained activation (4000–10,000). This demonstrates the OTL’s ability to produce output spikes via internal oscillator ring coupling and proteinoid–actin dynamics, rather than exclusively stimulus-driven feedforward pathways. Periodic vertical activation slices depict synchronized gate bursting under the control of underlying limit cycle regimes. The new temporal patterns suggest a complex interplay between the gates. The OTL acts as a decision-making unit. It combines the rapid switching of the input gates and the controlled output response. The heatmap shows how, over time, a balance of coordination and segregation of input sensing and intrinsic output spiking arises across the biocomposite interface.

The logic gate activation heatmap (Figure 26) demonstrates how erratic output spiking continues despite inert inputs across the proteinoid–actin interface. The underlying architecture appears to allow for bursts of coordinated high-intensity micro-events across input and output gates, as shown in the periodic vertical activation slices. To determine whether such phenomena represent global synchronization or localized micro-domain coordination, the logic fabric evolution must be cross-correlated with microscopic cytoskeletal rearrangement. This can reveal whether apparent spontaneous logic switches are due to structural changes such as filament rotations or density fluctuations. Overall, using the dynamic bio-interface as an adaptable logic processor gives a framework to decode emergent computing from the bottom–up.

## Discussion

We have designed a system of proteinoid microspheres spanned by actin filaments, called proteinoid–actin system. The complex microstructures observed in scanning electron micrographs of self-assembled proteinoid–actin composites provide compelling evidence of complex morphological emergence that exceeds mere random aggregations. As noted by distinguished biologist George Wald many years ago, certain molecular primordial soups possess inherent organizing properties encoded within their underlying physics, enabling them to spontaneously engage in lifelike architectural self-construction without the need for external biological machinery.^82^ Our current findings not only support the existence of these inherently exploitable “self-assembly” phenomena across different length scales, but also establish their presence in non-living polypeptides interfacing with cytoskeletal filaments. Through quantifying the shape landscapes formed by these assemblies, we can establish links between configurational free energy sinks and recurrently selected archetypal templates that facilitate efficient information propagation. Ultimately, by examining the relationships between molecular stacking forces, emergent curvatures, and computational utility, we can establish a crucial foundation for advancing dynamic biological fabrics into artificial cognition substrates.

We investigated the effects of driving the integrated proteinoid–actin system with chaotic rhythms on the modulation of extremes at the bio–abiotic interface. We observed that oscillatory inputs originating from various systems such as Logistic maps, Lorentz attractor, and Rössler attractor demonstrate a substantial regularization of voltage due to the composite structure. Quantitatively, the stability of the signals experiences significant improvement, resulting in the clamping of variations by more than 20 dB under certain circumstances. Here, “stability” means less noise and variability in the output signal. It does not refer to a fixed point in the phase space of the dynamical system. This use focuses on the system’s ability to suppress chaos and maintain signal coherence. Figures 8–22 showcase the quantitative characterizations of the clamping effect that occurs during the transmission of chaotic waveforms through the proteinoid–cytoskeletal interface. There has been a notable change in the average input voltage levels, transitioning from the initial values of approximately 0 V/–80 dB to confined ranges of 0/–100 dB. The significant 20 dB improvement in signal condensation underscores the proteinoid–cytoskeletal interface’s capacity to convert complex waveforms into a more succinct and controllable form. The data show a consistent pattern: chaotic biomolecular networks naturally transform into organized electrical signals without the need for external circuits. This observation of emergent signal regularization by bio-molecular matrices aligns with coordinated phenomena noted through independent efforts interfacing distinct chaos generating systems with specialized biocomposite formulations.^83,84^ In addition to steady-state statistics, the preserved frequency alignment confirms real-time monitoring, eliminating any delays between input and output motifs. Notably, it is observed that different chaotic drivers induce similar suppression, indicating the presence of common volatility containment mechanisms directed by materials. While the enhanced smoothness appears to mimic a filtering process, a closer examination reveals additional calculations enabled by cytoskeletal reconfiguration. Cross-correlation analysis reveals windows of output autonomy, suggesting the presence of complex intra-mesh logical operations despite the passive nature of the stimulus. To establish links between emergent conduction pathways and structural transitions in activation cascades, simultaneous multi-shot imaging techniques are necessary. The consistent and pronounced optimization of signals demonstrates the functional significance of the proteinoid–cytoskeletal network, which exhibits local disorder and global synchronization. The classification of the diverse logical representations made possible through tunable architectural couplings paves the way for the development of bio-inspired circuits that incorporate rational engineering design concepts into synthetic biological substrates. Furthermore, by integrating molecular tools for programmable self-construction with order templates derived from physical theory, the exploration of the mutually beneficial hybridization of biomolecular complexity and classical dynamical models is expected to continue.

The dynamic interplay between proteinoid structures and the actin network, driven by various chaotic rhythms, uncovers a consistent pattern in signal regularization within the bio-derived interface. Through quantitative analysis, we observe that the input Lyapunov exponents confirm the highly irregular waveforms generated by discrete logistic maps and multidimensional Rössler attractor trajectories, which exhibit exponential divergence amplification of small perturbations. However, the transmitted outputs demonstrate more negative exponent values, indicating enhanced suppression effects. The significant reduction in divergence rates, up to 17–fold, verifies the microscale coordination that actively constrains oscillations within well-defined boundaries despite external volatility. This improved smoothness, resembling linear filtering, is attributed to the productive modulation facilitated by proteinoid architectural adaptations and cytoskeletal network reconfigurations that collectively harness randomness. The emergence of orderly representations from underlying disorder positions these bio-composite networks as promising candidates for unreliable logic gate arrays that exploit noise. By interpreting the disorder-to-order transitions through the lens of cellular non-equilibrium thermodynamics, we establish a framework for bridging conservation principles with dissipative signaling, thereby contextualizing our observations. Generally, quantifying Lyapunov metrics provides a robust signature to classify modulation effects across dynamical regimes, paving the way for materials optimization that balances plasticity and robustness—two fundamental characteristics of biological computation.

The findings of our study indicate that the composite proteinoid–actin system displays several oscillatory phenomena, such as synchronization and phase-locking.^85,86^ Employing these features for unconventional computing^87^ is a promising method of utilizing the complex nature of biochemicals for information processing.^88−90^ The bio-composite oscillator networks represent a progression toward upcoming bio-inspired technologies, drawing inspiration from the adaptable dynamics observed in biological systems across many sizes.^91,92^

The application of the FitzHugh–Nagumo system, which emulates neuronal waveforms, provides further evidence of the activation responsiveness displayed by proteinoid architectures, akin to that observed in formal neurons. FitzHugh–Nagumo inputs are not essential for observing spiking dynamics. But, they are a valuable, biologically relevant model for probing the system’s behavior under neuron-like stimulation. Other input signals, like simple oscillatory or chaotic waveforms, may also evoke similar dynamics. But, the FitzHugh–Nagumo system ensures compatibility with bioinspired signal processing. Specifically, when examining the sigmoid coupling system, it becomes evident that subthreshold fluctuations result in V1out output variations that are directly proportional. However, surpassing the excitation threshold gives rise to rapid and distinct spiking profiles, characteristic of neuronal firing patterns. This regulatory behavior supports the notion that proteinoid microspheres possess excitability comparable to that of the nervous system, under the influence of specific chemical or electrical stimulation methods. Moreover, the maintained synchronicity in timing and spectral alignment affirms the real-time tracking capabilities of the integrated bio-composite interface. This highlights its ability to swiftly analyze and interpret signals, eschewing the need for slower processing methods. By extending the concept of excitability to activation cascades within networks, we can gain insights into the formation of coordinated logic gate motifs and the spontaneous emergence of ordered outcomes in interconnected heterogeneous architectures. Heatmap visualizations provide evidence that short periods of time exhibit synchronized bursting, involving sensory, transmission, and gating nodes simultaneously. Exploring whether these outbreaks lead to the synchronization of reorganization at a local or global scale within the composite structure can elucidate how molecular level changes influence computation patterns at the systems level. In summary, the diverse range of measured responses and excitability support the notion that the proteinoid–actin network possesses versatile signaling capacities. Pursuing optimization strategies for configurable logic operations and leveraging self-organized bio-molecular pathways for decision-making present exciting prospects to harness the principles of biological complexity in unconventional computing applications.

Overall, the results demonstrate that specialized nonlinear mechanisms play a crucial role in translating diverse external inputs into a structured and limited frequency response. The observed clamping effect and the significant shift in input voltage averages highlight the productive modulation functionality of the proteinoid–cytoskeletal interface. This interface effectively transforms chaotic waveforms into concise and manageable representations without the need for external shaping circuits.

The analysis of variance partitioning further elucidates the proteinoid architectural reconfigurations and cytoskeletal transitions that contribute to the transformation of unpredictable inputs into controlled and stable states. These reconfigurations and transitions work in conjunction to create cohesion and coherence between biomolecules, which ultimately affect the electrical transmission of the dynamic structure.

The presence of stable co-excitation at the nanosecond time scale and the absence of long anti-correlated regions indicate cohesive interactions between biomolecules, both in connected and disconnected proteinoid microstates. This cohesive behavior extends to locked filamentous regimes, reinforcing the notion of global coordination between the biological and non-living components of the system. Real-time microscopy research can further elucidate the link between reported response dynamics and structural changes at the proteinoid–actin biocomposite interface.

The nonlinear characteristics of the integrated platform are confirmed through similarities in input–output patterns. Moreover, the observation of intermittent decoupling and partially conductive versus insulating states suggests that proteinoid architectures undergo complex conformational changes under extreme driving conditions. These changes give rise to emergent nonlinearity, which governs the input–output signal changes observed.

The study also highlights the role of proteinoid architectural reconfigurations in selective filtering or convolution effects on upstream driving variables. The observed electrical modifications, including amplitude suppression and spectral alignment in the output, point to molecular reconfigurations mediated by the dynamic bio–abiotic interface.

The observed relationship between signal magnitudes and their standard deviations requires careful consideration. Our initial experiments found that standard deviations often exceeded peak magnitudes. This may raise doubts about the signal’s reliability. However, this variability shows an important trait of our proteinoid–actin system. It can actively process and regularize inputs. For example, in the Rössler oscillation experiments, the input signal had a high variability (std. dev. 2.46 V) compared to its mean (0.13 V). The output had a much lower variation (std. dev. 0.11 V, mean 0.01 V). This 20-fold drop in signal variance shows that the biocomposite network is an effective signal conditioner. The high initial standard deviations thus represent the complex, chaotic nature of our input signals rather than measurement uncertainty. The system’s consistent ability to reduce these variations across different input patterns (FitzHugh–Nagumo, Baker’s Map, Rössler) proves it can process signals reliably. This is despite the high variability in the input signals.

In summary, the results presented in this study shed light on the complex mechanisms by which the proteinoid–cytoskeletal interface modulates input signals. The findings have significant implications for the fields of signal processing and bio-inspired computing. More research is needed to understand the processes behind chaotic matter reconfiguration. We should explore its potential for bioinspired architectures.

## Conclusions

This study showcases the emergence of oscillatory events at the boundary of a composite system consisting of proteinoid microstructures combined with cytoskeletal actin networks. Our investigation discovered that the proteinoid–actin network has the ability to respond to a wide range of signals, from chaotic rhythms to rhythmic biosignals, in order to drive this dynamic bio–abiotic architecture.

To formalize our understanding of the system’s mechanistic behavior, we propose that its response to various chaotic input signals can provide insights into its computational principles. For example, simple periodic inputs could establish a baseline for the system’s dynamics. Chaotic inputs could reveal its ability to reduce noise, remain stable, and process nonlinearly. These observations could help build a theoretical model. It would identify critical parameters, like frequency coupling and amplitude modulation. These govern the network’s emergent behavior. They include trajectory convergence within phase space.

Our work shows we can process a single input signal. However, we need to test it more to prove we can do multi-input Boolean operations. Our early results with logistic map transformations show promise. But, scaling these networks for complex tasks poses challenges. These include: maintaining signal integrity across larger networks, ensuring reliable threshold behavior over multiple gates, and achieving consistent input–output relationships. Future work should focus on: (1) characterizing two-input logic operations, (2) developing protocols for network scaling, and (3) creating metrics for reliability in larger proteinoid–actin assemblies.

## Data Availability

This data is accessible via the online database Zenodo (https://zenodo.org/records/10616320).
